# Supplementary material for: A comprehensive preclinical study supporting clinical trial of oncolytic chimeric poxvirus CF33-hNIS-anti-PD-L1 to treat breast cancer
Source: Mol Ther Methods Clin Dev. 2021 Dec 6;24:102–16. doi: 10.1016/j.omtm.2021.12.002 (PMC8718831; doi:10.1016/j.omtm.2021.12.002)
Supplement: Document S1. Tables S1–S10 [file mmc1.pdf]

OMTM, Volume *24*

Supplemental information

A comprehensive preclinical study supporting clinical trial of oncolytic chimeric poxvirus CF33-hNIS-anti-PD-L1 to treat breast cancer

Shyambabu Chaurasiya, Annie Yang, Zhifang Zhang, Jianming Lu, Hannah Valencia, Sang-In Kim, Yanghee Woo, Suanne G. Warner, Tove Olafsen, Yuqi Zhao, Xiwei Wu, Seymour Fein, Linda Cheng, Maria Cheng, Nicholas Ede, and Yuman Fong

Table 1: List of CF33 genes involved in entry, attachment, replication and pathogenesis, and their origins.

Categories	Descriptions	Genes	Origins
Prevention of superinfection	prevents superinfection	A33R	AS
	prevents superinfection	A36R	WR or Rabbitpox
Precursor Metabolism	dNTP synthesis	F4L	IHD
	dNTP synthesis	A48R	IHD
	dNTP synthesis	I4L	WR
	dNTP synthesis	J2R	WR or IHD or Rabbitpox
Pathogenesis	Semaphorin-like	A39R	AS
	inhibitor of antigen presentation by MHC II	A35R	AS or Lister or Rabbitpox
	complement control	C21L	CL or WR or IHD or Lister or Rabbitpox
	complement control	C21L	CL or WR or IHD or Lister or Rabbitpox
	TLR inhibitor	A46R	IHD
	TLR inhibitor	A52R	IHD
	anti-apoptotic protein serpin	SPI-2/CrmA	IHD
	Type 1 IFN-R mimic	B18R	IHD
	inhibits IL-18	C12L	IHD
	eIF2a-mimic; IFN I resistance	K3L	Lederle
	Inhibition of NF-B and IRF3	K7R	Lederle
	Inhibition of apoptosis	N1L	Lederle or WR or IHD or Lister or Rabbitpox
	EGF-like growth factor	C11R	WR
	Type 2 IFN-R mimic	B8R	WR or IHD
	dsRNA binding protein; block IFN I	E3L	WR or IHD or Rabbitpox
Entry	Entry	A21L	AS or IHD or Lister or Rabbitpox
	Entry	G3L	Lederle or AS or WR or IHD or Lister or Rabbitpox
	Entry	H2R	Lederle or AS or WR or Lister
	Entry	A28L	Lederle or WR
	Entry	F9L	Lederle or WR or IHD or Lister
	Entry	L5R	Lederle or WR or IHD or Lister
	Entry	I2L	Lister
	Entry	J5L	Lister
	Entry	A16L	Rabbitpox
	Entry	G9R	WR
	Entry	L1R	WR
DNA replication	FEN1 like nuclease	G5R	AS
	DNA polymerase	E9L	AS or IHD or Lister or Rabbitpox
	Telomere binding protein 1	I1L	AS or Lister
	Processivity factor	A20R	AS or Lister
	ATPase	A32L	IHD
	DNA ligase	A50R	IHD
	Protein Kinase	B1R	IHD
	Uracil. DNA glkycosylase	D4R	Lederle or WR

Table 1 (Continued):

DNA replication	Topoisomerase	H6R	Rabbitpox
	Single stranded DNA binding protein	I3L	WR
	Helicase-Primase	D5R	WR
	Holiday junction resolvase	A22R	WR
Cell-cell fusion	cell-cell fusion	A56R	IHD
	cell-cell fusion	K2L/SPI-3	WR
Attachment	Attachment	A26L	Lister
	Attachment	H3L	WR
	Attachment	D8L	WR
	Attachment	A27L	WR

Table 2: Summary of Study Design – Toxicology Groups

Group	Number of Mice	Animal Number (M/F)	Tumor Induction	Test Article Treatment		In-life Procedure	Termination	Terminal Procedure
1	6M /6F	101-106 /151-156	Yes	IT 1X Day 0	Control Article (Vehicle)	Clinical Observations: 1x daily; Body Condition Scoring: weekly; Body Weights: weekly; Food Consumption: weekly; Tumor measurement: 2x weekly	24 Hr	Clinical pathology; Necropsy; organ weights; histopathology
	6M /6F	107-112 /157-162					Day 7	
	8M /8F	113-120 /163-170					Day 21	
2	6M /6F	201-206 /251-256			CF33-hNIS-antiPDL1 1E03 PFU		24 Hr	
	6M /6F	207-212 /257-262					Day 7	
	8M /8F	213-220 /263-270					Day 21	
3	6M /6F	301-306 /351-356			CF33-hNIS-antiPDL1 1E04 PFU		24 Hr	
	6M /6F	307-312 /357-362					Day 7	
	8M /8F	313-320 /363-370					Day 21	
4	6M /6F	401-406 /451-456			CF33-hNIS-antiPDL1 1E05 PFU		24 Hr	
	6M /6F	407-412 /457-462					Day 7	
	8M /8F	413-420 /463-470					Day 21	
5	6M /6F	501-506 /551-556		IT 6X Day 0-12	Control Article (Vehicle)		Day 12	
	8M /8F	507-514 /557-564					Day 21	
6	6M /6F	601-606 /651-656			CF33-hNIS-antiPDL1 1E03 PFU		Day 12	
	8M /8F	607-614 /657-664					Day 21	
7	6M /6F	701-706 /751-756			CF33-hNIS-antiPDL1 1E05 PFU		Day 12	
	8M /8F	707-714 /757-764					Day 21	

Table 3: Summary of Study Design – Biodistribution Groups

Group	Number of Mice	Animal Number (F)	Tumor Induction	Test Article Treatment		In-life Procedure	Termination	Terminal Procedure
**8**	5F	851-855	Yes	IT 1X Day0	Control Article (Vehicle)	Clinical Observations: 1x daily; Body Condition Scoring: weekly; Body Weights: weekly; Food Consumption: weekly; Tumor measurement: 2x weekly	Day 7	Terminal blood and tissue collection for viral plaques and PCR
	8F	856-863					Day 21	
**9**	5F	951-955			CF33-hNIS-antiPDL1 1E03 PFU		Day 7	
	8F	956-963					Day 21	
**10**	5F	1051-1055			CF33-hNIS-antiPDL1 1E04 PFU		Day 7	
	8F	1056-1063					Day 21	
**11**	5F	1151-1155			CF33-hNIS-antiPDL1 1E05 PFU		Day 7	
	8F	1156-1163					Day 21	
**12**	5F	1251-1255		IT 6X Day 0-12	Control Article (Vehicle)		Day 12	
	8F	1256-1263			0.1 mL		Day 21	
**13**	5F	1351-1355			CF33-hNIS-antiPDL1 1E03 PFU		Day 12	
	8F	1356-1363					Day 21	
**14**	5F	1451-1455			CF33-hNIS-antiPDL1 1E05 PFU		Day 12	
	8F	1456-1463					Day 21	

Table 4: Summary of Study Design – Troponin Groups

Group	Number of Mice	Animal Number (M/F)	Tumor Induction	Test Article Treatment		In-life Procedure	Termination	Terminal Procedure
**15**	5F	1551-1555	Yes	IT 1X Day0	Control Article (Vehicle)	Clinical Observations: 1x daily; Body Condition Scoring: weekly; Body Weights: weekly; Food Consumption: weekly; Tumor measurement: 2x weekly	24 Hr	Terminal blood collection for troponins
	8F	1556-1563					Day 14	
**16**	5F	1651-1655			CF33-hNIS-antiPDL1 1E05 PFU		24 Hr	
	8F	1656-1663					Day 14	
**17**	8F	1751-1758		IT 6X Day 0-12	Control Article (Vehicle)		Day 21	
**18**	8F	1851-1858			CF33-hNIS-antiPDL1 1E03 PFU			
**19**	8F	1851-1858			CF33-hNIS-antiPDL1 1E05 PFU			

Table 5: Summary of Study Design – Cytokine Groups

Group	Number of Mice	Animal Number (M/F)	Tumor Induction	Test Article Treatment		In-life Procedure	Termination	Terminal Procedure
	5F	2051-2055			Control Article (Vehicle)		3 Hr	
**20**	5F	2056-2060					Day 7	
	8F	2061-2068					Day 14	
	5F	2151-2155		IT 1X Day0	CF33-hNIS-antiPDL1 1E03 PFU	Clinical Observations: 1x daily; Body Condition Scoring: weekly; Body Weights: weekly; Food Consumption: weekly; Tumor measurement: 2x weekly	3 Hr	
**21**	5F	2156-2160					Day 7	
	8F	2161-2168					Day 14	
	5F	2251-2255			CF33-hNIS-antiPDL1 1E03 PFU		3 Hr	
**22**	5F	2256-2260					Day 7	Terminal blood collection for cytokines
	8F	2261-2268	Yes				Day 14	
**23**	8F	2351-2358		IT 6X Day 0-12	Control Article (Vehicle)			
**24**	8F	2451-2458			CF33-hNIS-antiPDL1 1E03 PFU		Day 21	
**25**	8F	2551-2558			CF33-hNIS-antiPDL1 1E05 PFU			

Table 6: Sample Concentrations of IFN-γ in Mouse Serum

Run ID	Subject	Day (Nominal)	Concentration (pg/mL)	Concentration Units	Dilution Factor	Biological Matrix
36	2051	3 Hr	0.448	pg/mL	1	Serum
36	2052	3 Hr	BLQ<(0.187)	pg/mL	1	Serum
36	2053	3 Hr	BLQ<(0.187)	pg/mL	1	Serum
36	2054	3 Hr	BLQ<(0.187)	pg/mL	1	Serum
36	2055	3 Hr	BLQ<(0.187)	pg/mL	1	Serum
36	2056	7	BLQ<(0.187)	pg/mL	1	Serum
36	2057	7	0.313	pg/mL	1	Serum
36	2058	7	BLQ<(0.187)	pg/mL	1	Serum
36	2059	7	0.316	pg/mL	1	Serum
36	2060	7	0.228	pg/mL	1	Serum
36	2061	14	0.31	pg/mL	1	Serum
36	2062	14	0.32	pg/mL	1	Serum
36	2063	14	0.498	pg/mL	1	Serum
36	2064	14	0.639	pg/mL	1	Serum
36	2065	14	0.625	pg/mL	1	Serum
36	2066	14	0.434	pg/mL	1	Serum
36	2067	14	0.222	pg/mL	1	Serum
36	2068	14	0.384	pg/mL	1	Serum
36	2151	3 Hr	0.331	pg/mL	1	Serum
36	2152	3 Hr	0.207	pg/mL	1	Serum
36	2153	3 Hr	0.316	pg/mL	1	Serum
36	2154	3 Hr	BLQ<(0.187)	pg/mL	1	Serum
36	2155	3 Hr	0.187	pg/mL	1	Serum
36	2156	7	0.39	pg/mL	1	Serum
36	2157	7	BLQ<(0.187)	pg/mL	1	Serum
36	2158	7	0.365	pg/mL	1	Serum
36	2159	7	0.23	pg/mL	1	Serum
36	2160	7	0.217	pg/mL	1	Serum
36	2161	14	0.28	pg/mL	1	Serum
36	2162	14	0.26	pg/mL	1	Serum
36	2163	14	0.308	pg/mL	1	Serum
36	2164	14	BLQ<(0.187)	pg/mL	1	Serum
36	2165	14	0.481	pg/mL	1	Serum
36	2166	14	0.328	pg/mL	1	Serum
36	2167	14	0.35	pg/mL	1	Serum
41	2168	14	0.432	pg/mL	1	Serum
41	2251	3 Hr	0.203	pg/mL	1	Serum
41	2252	3 Hr	0.395	pg/mL	1	Serum
41	2253	3 Hr	BLQ<(0.187)	pg/mL	1	Serum
41	2254	3 Hr	0.279	pg/mL	1	Serum
41	2255	3 Hr	0.212	pg/mL	1	Serum

41	2256	7	0.231	pg/mL	1	Serum
41	2257	7	0.944	pg/mL	1	Serum

Table 6 (Continued)

41	2258	7	0.344	pg/mL	1	Serum
41	2259	7	0.381	pg/mL	1	Serum
41	2260	7	0.202	pg/mL	1	Serum
41	2261	14	0.401	pg/mL	1	Serum
41	2262	14	0.353	pg/mL	1	Serum
41	2263	14	0.298	pg/mL	1	Serum
41	2264	14	0.58	pg/mL	1	Serum
41	2265	14	0.207	pg/mL	1	Serum
41	2266	14	BLQ<(0.187)	pg/mL	1	Serum
41	2267	14	0.244	pg/mL	1	Serum
41	2268	14	0.401	pg/mL	1	Serum
41	2351	19	BLQ<(0.187)	pg/mL	1	Serum
41	2352	19	BLQ<(0.187)	pg/mL	1	Serum
41	2353	19	BLQ<(0.187)	pg/mL	1	Serum
41	2354	19	0.195	pg/mL	1	Serum
41	2355	14	0.874	pg/mL	1	Serum
41	2356	19	0.201	pg/mL	1	Serum
41	2357	21	0.215	pg/mL	1	Serum
41	2358	19	0.291	pg/mL	1	Serum
41	2451	19	BLQ<(0.187)	pg/mL	1	Serum
41	2452	14	0.314	pg/mL	1	Serum
41	2453	21	0.239	pg/mL	1	Serum
41	2454	14	0.273	pg/mL	1	Serum
41	2455	14	0.207	pg/mL	1	Serum
41	2456	14	0.326	pg/mL	1	Serum
41	2457	19	0.319	pg/mL	1	Serum
41	2458	14	0.309	pg/mL	1	Serum
31	2551	19	BLQ<(0.187)	pg/mL	1	Serum
31	2552	19	BLQ<(0.187)	pg/mL	1	Serum
31	2553	14	0.261	pg/mL	1	Serum
31	2554	19	0.196	pg/mL	1	Serum
31	2555	19	BLQ<(0.187)	pg/mL	1	Serum
31	2556	21	0.282	pg/mL	1	Serum
31	2557	19	BLQ<(0.187)	pg/mL	1	Serum
31	2558	21	0.296	pg/mL	1	Serum

Table 7: Sample Concentrations of IL-1β in Mouse Serum

Run ID	Subject	Day Nominal	Concentration (pg/mL)	Concentration Units	Dilution Factor	Biological Matrix
37	2051	3 Hr	BLQ<(0.374)	pg/mL	1	Serum
37	2052	3 Hr	BLQ<(0.374)	pg/mL	1	Serum
37	2053	3 Hr	BLQ<(0.374)	pg/mL	1	Serum
37	2054	3 Hr	BLQ<(0.374)	pg/mL	1	Serum
37	2055	3 Hr	BLQ<(0.374)	pg/mL	1	Serum
37	2056	7	BLQ<(0.374)	pg/mL	1	Serum
37	2057	7	BLQ<(0.374)	pg/mL	1	Serum
37	2058	7	BLQ<(0.374)	pg/mL	1	Serum
37	2059	7	BLQ<(0.374)	pg/mL	1	Serum
37	2060	7	BLQ<(0.374)	pg/mL	1	Serum
37	2061	14	BLQ<(0.374)	pg/mL	1	Serum
37	2062	14	BLQ<(0.374)	pg/mL	1	Serum
37	2063	14	BLQ<(0.374)	pg/mL	1	Serum
37	2064	14	BLQ<(0.374)	pg/mL	1	Serum
37	2065	14	BLQ<(0.374)	pg/mL	1	Serum
37	2066	14	BLQ<(0.374)	pg/mL	1	Serum
37	2067	14	BLQ<(0.374)	pg/mL	1	Serum
37	2068	14	BLQ<(0.374)	pg/mL	1	Serum
37	2151	3 Hr	BLQ<(0.374)	pg/mL	1	Serum
37	2152	3 Hr	BLQ<(0.374)	pg/mL	1	Serum
37	2153	3 Hr	BLQ<(0.374)	pg/mL	1	Serum
37	2154	3 Hr	BLQ<(0.374)	pg/mL	1	Serum
37	2155	3 Hr	BLQ<(0.374)	pg/mL	1	Serum
37	2156	7	BLQ<(0.374)	pg/mL	1	Serum
37	2157	7	BLQ<(0.374)	pg/mL	1	Serum
37	2158	7	BLQ<(0.374)	pg/mL	1	Serum
37	2159	7	BLQ<(0.374)	pg/mL	1	Serum
37	2160	7	BLQ<(0.374)	pg/mL	1	Serum
37	2161	14	BLQ<(0.374)	pg/mL	1	Serum
37	2162	14	BLQ<(0.374)	pg/mL	1	Serum
37	2163	14	BLQ<(0.374)	pg/mL	1	Serum
37	2164	14	BLQ<(0.374)	pg/mL	1	Serum
37	2165	14	BLQ<(0.374)	pg/mL	1	Serum
37	2166	14	BLQ<(0.374)	pg/mL	1	Serum
37	2167	14	BLQ<(0.374)	pg/mL	1	Serum
42	2168	14	BLQ<(0.374)	pg/mL	1	Serum
42	2251	3 Hr	BLQ<(0.374)	pg/mL	1	Serum
42	2252	3 Hr	BLQ<(0.374)	pg/mL	1	Serum
42	2253	3 Hr	BLQ<(0.374)	pg/mL	1	Serum
42	2254	3 Hr	BLQ<(0.374)	pg/mL	1	Serum
42	2255	3 Hr	BLQ<(0.374)	pg/mL	1	Serum

42	2256	7	BLQ<(0.374)	pg/mL	1	Serum

Table 7. (continued)

42	2257	7	BLQ<(0.374)	pg/mL	1	Serum
42	2258	7	BLQ<(0.374)	pg/mL	1	Serum
42	2259	7	BLQ<(0.374)	pg/mL	1	Serum
42	2260	7	BLQ<(0.374)	pg/mL	1	Serum
42	2261	14	BLQ<(0.374)	pg/mL	1	Serum
42	2262	14	BLQ<(0.374)	pg/mL	1	Serum
42	2263	14	BLQ<(0.374)	pg/mL	1	Serum
42	2264	14	BLQ<(0.374)	pg/mL	1	Serum
42	2265	14	BLQ<(0.374)	pg/mL	1	Serum
42	2266	14	BLQ<(0.374)	pg/mL	1	Serum
42	2267	14	BLQ<(0.374)	pg/mL	1	Serum
42	2268	14	BLQ<(0.374)	pg/mL	1	Serum
42	2351	19	BLQ<(0.374)	pg/mL	1	Serum
42	2352	19	BLQ<(0.374)	pg/mL	1	Serum
42	2353	19	BLQ<(0.374)	pg/mL	1	Serum
42	2354	19	BLQ<(0.374)	pg/mL	1	Serum
42	2355	14	BLQ<(0.374)	pg/mL	1	Serum
42	2356	19	BLQ<(0.374)	pg/mL	1	Serum
42	2357	21	BLQ<(0.374)	pg/mL	1	Serum
42	2358	19	BLQ<(0.374)	pg/mL	1	Serum
42	2451	19	BLQ<(0.374)	pg/mL	1	Serum
42	2452	14	BLQ<(0.374)	pg/mL	1	Serum
42	2453	21	BLQ<(0.374)	pg/mL	1	Serum
42	2454	14	BLQ<(0.374)	pg/mL	1	Serum
42	2455	14	BLQ<(0.374)	pg/mL	1	Serum
42	2456	14	BLQ<(0.374)	pg/mL	1	Serum
42	2457	19	BLQ<(0.374)	pg/mL	1	Serum
42	2458	14	BLQ<(0.374)	pg/mL	1	Serum
32	2551	19	BLQ<(0.374)	pg/mL	1	Serum
32	2552	19	BLQ<(0.374)	pg/mL	1	Serum
32	2553	14	BLQ<(0.374)	pg/mL	1	Serum
32	2554	19	BLQ<(0.374)	pg/mL	1	Serum
32	2555	19	BLQ<(0.374)	pg/mL	1	Serum
32	2556	21	BLQ<(0.374)	pg/mL	1	Serum
32	2557	19	BLQ<(0.374)	pg/mL	1	Serum
32	2558	21	BLQ<(0.374)	pg/mL	1	Serum

Table 8: Sample Concentrations of IL-6 in Mouse Serum

Run ID	Subject	Day (Nominal)	Concentration (pg/mL)	Concentration Units	Dilution Factor	Biological Matrix
38	2051	3 Hr	8.06	pg/mL	1	Serum
38	2052	3 Hr	3.23	pg/mL	1	Serum
38	2053	3 Hr	1.96	pg/mL	1	Serum
38	2054	3 Hr	2.86	pg/mL	1	Serum
38	2055	3 Hr	11.9	pg/mL	1	Serum
38	2056	7	6.21	pg/mL	1	Serum
38	2057	7	13.8	pg/mL	1	Serum
38	2058	7	7.01	pg/mL	1	Serum
38	2059	7	9.59	pg/mL	1	Serum
38	2060	7	28.4	pg/mL	1	Serum
38	2061	14	20.6	pg/mL	1	Serum
38	2062	14	37.6	pg/mL	1	Serum
38	2063	14	27.7	pg/mL	1	Serum
38	2064	14	25.6	pg/mL	1	Serum
38	2065	14	20.1	pg/mL	1	Serum
38	2066	14	89.1	pg/mL	1	Serum
38	2067	14	2.52	pg/mL	1	Serum
38	2068	14	33.8	pg/mL	1	Serum
38	2151	3 Hr	4.74	pg/mL	1	Serum
38	2152	3 Hr	4.21	pg/mL	1	Serum
38	2153	3 Hr	2.54	pg/mL	1	Serum
38	2154	3 Hr	6.09	pg/mL	1	Serum
38	2155	3 Hr	3.14	pg/mL	1	Serum
38	2156	7	13.2	pg/mL	1	Serum
38	2157	7	14.3	pg/mL	1	Serum
38	2158	7	9.41	pg/mL	1	Serum
38	2159	7	27.4	pg/mL	1	Serum
38	2160	7	7.58	pg/mL	1	Serum
38	2161	14	27.2	pg/mL	1	Serum
38	2162	14	50.7	pg/mL	1	Serum
38	2163	14	90.1	pg/mL	1	Serum
38	2164	14	23.2	pg/mL	1	Serum
38	2165	14	28.5	pg/mL	1	Serum
38	2166	14	75.3	pg/mL	1	Serum
38	2167	14	34.8	pg/mL	1	Serum
43	2168	14	100	pg/mL	1	Serum
43	2251	3 Hr	3.57	pg/mL	1	Serum
43	2252	3 Hr	12.6	pg/mL	1	Serum
43	2253	3 Hr	4.22	pg/mL	1	Serum
43	2254	3 Hr	4.48	pg/mL	1	Serum

43	2255	3 Hr	4.3	pg/mL	1	Serum

Table 8. (continued)

43	2256	7	9.93	pg/mL	1	Serum
43	2257	7	17.1	pg/mL	1	Serum
43	2258	7	57.8	pg/mL	1	Serum
43	2259	7	10.3	pg/mL	1	Serum
43	2260	7	7.88	pg/mL	1	Serum
43	2261	14	27.6	pg/mL	1	Serum
43	2262	14	187	pg/mL	1	Serum
43	2263	14	15.6	pg/mL	1	Serum
43	2264	14	34.9	pg/mL	1	Serum
43	2265	14	31.2	pg/mL	1	Serum
43	2266	14	14.2	pg/mL	1	Serum
43	2267	14	18.9	pg/mL	1	Serum
43	2268	14	25.2	pg/mL	1	Serum
43	2351	19	78.1	pg/mL	1	Serum
43	2352	19	30.8	pg/mL	1	Serum
43	2353	19	41.4	pg/mL	1	Serum
43	2354	19	28.7	pg/mL	1	Serum
43	2355	14	205	pg/mL	1	Serum
43	2356	19	89.2	pg/mL	1	Serum
43	2357	21	14.5	pg/mL	1	Serum
43	2358	19	221	pg/mL	1	Serum
43	2451	19	58.9	pg/mL	1	Serum
43	2452	14	21.9	pg/mL	1	Serum
43	2453	21	36.3	pg/mL	1	Serum
43	2454	14	56.6	pg/mL	1	Serum
43	2455	14	39.2	pg/mL	1	Serum
43	2456	14	20.8	pg/mL	1	Serum
43	2457	19	89.5	pg/mL	1	Serum
43	2458	14	33.3	pg/mL	1	Serum
33	2551	19	18.6	pg/mL	1	Serum
33	2552	19	47	pg/mL	1	Serum
33	2553	14	27.2	pg/mL	1	Serum
33	2554	19	33.4	pg/mL	1	Serum
33	2555	19	103	pg/mL	1	Serum
33	2556	21	43.4	pg/mL	1	Serum
33	2557	19	34.8	pg/mL	1	Serum
33	2558	21	6.91	pg/mL	1	Serum

Table 9: Sample Concentrations of IL-10 in Mouse Serum

Run ID	Subject	Day (Nominal)	Concentration (pg/mL)	Concentration Units	Dilution Factor	Biological Matrix
39	2051	3 Hr	7.37	pg/mL	1	Serum
39	2052	3 Hr	2.17	pg/mL	1	Serum
39	2053	3 Hr	2.61	pg/mL	1	Serum
39	2054	3 Hr	3.01	pg/mL	1	Serum
39	2055	3 Hr	3.78	pg/mL	1	Serum
39	2056	7	3.67	pg/mL	1	Serum
39	2057	7	3.89	pg/mL	1	Serum
39	2058	7	2.99	pg/mL	1	Serum
39	2059	7	4.72	pg/mL	1	Serum
39	2060	7	4.21	pg/mL	1	Serum
39	2061	14	5.46	pg/mL	1	Serum
39	2062	14	6.54	pg/mL	1	Serum
39	2063	14	7.39	pg/mL	1	Serum
39	2064	14	4.79	pg/mL	1	Serum
39	2065	14	6.29	pg/mL	1	Serum
39	2066	14	14.5	pg/mL	1	Serum
39	2067	14	3.34	pg/mL	1	Serum
39	2068	14	7.95	pg/mL	1	Serum
39	2151	3 Hr	4.31	pg/mL	1	Serum
39	2152	3 Hr	3.34	pg/mL	1	Serum
39	2153	3 Hr	3.45	pg/mL	1	Serum
39	2154	3 Hr	1.49	pg/mL	1	Serum
39	2155	3 Hr	1.99	pg/mL	1	Serum
39	2156	7	4.27	pg/mL	1	Serum
39	2157	7	3.57	pg/mL	1	Serum
39	2158	7	4.44	pg/mL	1	Serum
39	2159	7	4	pg/mL	1	Serum
39	2160	7	3.21	pg/mL	1	Serum
39	2161	14	7.59	pg/mL	1	Serum
39	2162	14	8.29	pg/mL	1	Serum
39	2163	14	159	pg/mL	1	Serum
39	2164	14	4.62	pg/mL	1	Serum
39	2165	14	8.26	pg/mL	1	Serum
39	2166	14	8.82	pg/mL	1	Serum
39	2167	14	9.16	pg/mL	1	Serum
44	2168	14	3.96	pg/mL	1	Serum
44	2251	3 Hr	3.04	pg/mL	1	Serum
44	2252	3 Hr	4.08	pg/mL	1	Serum
44	2253	3 Hr	2.29	pg/mL	1	Serum
44	2254	3 Hr	2.2	pg/mL	1	Serum

44	2255	3 Hr	2.05	pg/mL	1	Serum
44	2256	7	4.58	pg/mL	1	Serum

Table 9. continued

44	2257	7	35.7	pg/mL	1	Serum
44	2258	7	6.64	pg/mL	1	Serum
44	2259	7	3.44	pg/mL	1	Serum
44	2260	7	3.03	pg/mL	1	Serum
44	2261	14	12.1	pg/mL	1	Serum
44	2262	14	11.8	pg/mL	1	Serum
44	2263	14	4.74	pg/mL	1	Serum
44	2264	14	5.75	pg/mL	1	Serum
44	2265	14	4.55	pg/mL	1	Serum
44	2266	14	2.12	pg/mL	1	Serum
44	2267	14	4.71	pg/mL	1	Serum
44	2268	14	5.29	pg/mL	1	Serum
44	2351	19	16.9	pg/mL	1	Serum
44	2352	19	9.97	pg/mL	1	Serum
44	2353	19	6.54	pg/mL	1	Serum
44	2354	19	4.82	pg/mL	1	Serum
44	2355	14	15.3	pg/mL	1	Serum
44	2356	19	11.5	pg/mL	1	Serum
44	2357	21	2.29	pg/mL	1	Serum
44	2358	19	25.8	pg/mL	1	Serum
44	2451	19	37.6	pg/mL	1	Serum
44	2452	14	9.18	pg/mL	1	Serum
44	2453	21	6.19	pg/mL	1	Serum
44	2454	14	10.1	pg/mL	1	Serum
44	2455	14	11.9	pg/mL	1	Serum
44	2456	14	11.5	pg/mL	1	Serum
44	2457	19	14.7	pg/mL	1	Serum
44	2458	14	7.13	pg/mL	1	Serum
34	2551	19	12	pg/mL	1	Serum
34	2552	19	8.77	pg/mL	1	Serum
34	2553	14	5.85	pg/mL	1	Serum
34	2554	19	8.38	pg/mL	1	Serum
34	2555	19	14.2	pg/mL	1	Serum
34	2556	21	7.43	pg/mL	1	Serum
34	2557	19	11.2	pg/mL	1	Serum
34	2558	21	2.65	pg/mL	1	Serum

Table 10: Sample Concentrations of TNF-α in Mouse Serum

Run ID	Subject	Day (Nominal)	Concentration (pg/mL)	Concentration Units	Dilution Factor	Biological Matrix
40	2051	3 Hr	8.07	pg/mL	1	Serum
40	2052	3 Hr	4.04	pg/mL	1	Serum
40	2053	3 Hr	3.21	pg/mL	1	Serum
40	2054	3 Hr	5.72	pg/mL	1	Serum
40	2055	3 Hr	3.71	pg/mL	1	Serum
40	2056	7	6.34	pg/mL	1	Serum
40	2057	7	7.61	pg/mL	1	Serum
40	2058	7	4.16	pg/mL	1	Serum
40	2059	7	7.49	pg/mL	1	Serum
40	2060	7	9.22	pg/mL	1	Serum
40	2061	14	13.4	pg/mL	1	Serum
40	2062	14	10.3	pg/mL	1	Serum
40	2063	14	12.2	pg/mL	1	Serum
40	2064	14	18.3	pg/mL	1	Serum
40	2065	14	15.8	pg/mL	1	Serum
40	2066	14	29	pg/mL	1	Serum
40	2067	14	4.01	pg/mL	1	Serum
40	2068	14	22.3	pg/mL	1	Serum
40	2151	3 Hr	5.36	pg/mL	1	Serum
40	2152	3 Hr	3.67	pg/mL	1	Serum
40	2153	3 Hr	3.66	pg/mL	1	Serum
40	2154	3 Hr	2.36	pg/mL	1	Serum
40	2155	3 Hr	3.58	pg/mL	1	Serum
40	2156	7	8.99	pg/mL	1	Serum
40	2157	7	5.21	pg/mL	1	Serum
40	2158	7	7.72	pg/mL	1	Serum
40	2159	7	8.31	pg/mL	1	Serum
40	2160	7	5.97	pg/mL	1	Serum
40	2161	14	16.6	pg/mL	1	Serum
40	2162	14	19.6	pg/mL	1	Serum
40	2163	14	60	pg/mL	1	Serum
40	2164	14	13	pg/mL	1	Serum
40	2165	14	13	pg/mL	1	Serum
40	2166	14	24.8	pg/mL	1	Serum
40	2167	14	21	pg/mL	1	Serum
45	2168	14	16.1	pg/mL	1	Serum
45	2251	3 Hr	3.69	pg/mL	1	Serum
45	2252	3 Hr	9.65	pg/mL	1	Serum

45	2253	3 Hr	3.94	pg/mL	1	Serum
45	2254	3 Hr	3.41	pg/mL	1	Serum
45	2255	3 Hr	2.57	pg/mL	1	Serum
45	2256	7	9.93	pg/mL	1	Serum

Table 10. (continued)

45	2257	7	26.5	pg/mL	1	Serum
45	2258	7	10.4	pg/mL	1	Serum
45	2259	7	7.86	pg/mL	1	Serum
45	2260	7	4.06	pg/mL	1	Serum
45	2261	14	26.4	pg/mL	1	Serum
45	2262	14	40.4	pg/mL	1	Serum
45	2263	14	17.9	pg/mL	1	Serum
45	2264	14	18.7	pg/mL	1	Serum
45	2265	14	12.5	pg/mL	1	Serum
45	2266	14	9.24	pg/mL	1	Serum
45	2267	14	8.69	pg/mL	1	Serum
45	2268	14	15	pg/mL	1	Serum
45	2351	19	49.6	pg/mL	1	Serum
45	2352	19	38.9	pg/mL	1	Serum
45	2353	19	22.7	pg/mL	1	Serum
45	2354	19	19.9	pg/mL	1	Serum
45	2355	14	30.9	pg/mL	1	Serum
45	2356	19	21.1	pg/mL	1	Serum
45	2357	21	6.95	pg/mL	1	Serum
45	2358	19	58.8	pg/mL	1	Serum
45	2451	19	25.8	pg/mL	1	Serum
45	2452	14	23	pg/mL	1	Serum
45	2453	21	15.3	pg/mL	1	Serum
45	2454	14	31.3	pg/mL	1	Serum
45	2455	14	29.4	pg/mL	1	Serum
45	2456	14	30.3	pg/mL	1	Serum
45	2457	19	43.2	pg/mL	1	Serum
45	2458	14	16.9	pg/mL	1	Serum
35	2551	19	21.4	pg/mL	1	Serum
35	2552	19	21.7	pg/mL	1	Serum
35	2553	14	17.8	pg/mL	1	Serum
35	2554	19	15.5	pg/mL	1	Serum
35	2555	19	24.3	pg/mL	1	Serum
35	2556	21	19.8	pg/mL	1	Serum
35	2557	19	24.1	pg/mL	1	Serum
35	2558	21	3.93	pg/mL	1	Serum
