# Supplementary material for: A comprehensive preclinical study supporting clinical trial of oncolytic chimeric poxvirus CF33-hNIS-anti-PD-L1 to treat breast cancer
Source: Mol Ther Methods Clin Dev. 2021 Dec 6;24:102–16. doi: 10.1016/j.omtm.2021.12.002 (PMC8718831; doi:10.1016/j.omtm.2021.12.002)
Supplement: Document S2. Article plus supplemental information [file mmc3.pdf]

Molecular Therapy
Methods & Clinical Development
Original Article

A comprehensive preclinical study supporting clinical trial of oncolytic chimeric poxvirus CF33-hNIS-anti-PD-L1 to treat breast cancer

Shyambabu Chaurasiya,[1] Annie Yang,[1] Zhifang Zhang,[1] Jianming Lu,[1] Hannah Valencia,[1] Sang-In Kim,[1] Yanghee Woo,[1] Suanne G. Warner,[2] Tove Olafsen,[3] Yuqi Zhao,[4] Xiwei Wu,[4] Seymour Fein,[5] Linda Cheng,[5] Maria Cheng,[5] Nicholas Ede,[6] and Yuman Fong[1]

[1]Department of Surgery, City of Hope National Medical Center, Familian Science building, Room#1100 1500 E Duarte Road, Duarte, CA 91010, USA; [2]Department of Surgery, Mayo Clinic, Rochester, MN 55902, USA; [3]Small Animal Imaging Core, Shared Resources, City of Hope National Medical Center, Duarte, CA 91010, USA; [4]Integrative Genomics Core, City of Hope National Medical Center, Duarte, CA 91010, USA; [5]CNF Pharma LLC, New City, NY 10956, USA; [6]Imugene Ltd, Sydney, NSW 2000, Australia

CF33-hNIS-anti-PD-L1 is an oncolytic chimeric poxvirus encoding two transgenes: human sodium iodide symporter and a single-chain variable fragment against PD-L1. Comprehensive preclinical pharmacology studies encompassing primary and secondary pharmacodynamics and biodistribution and safety studies were performed to support the clinical development of CF33-hNIS-anti-PD-L1. Most of the studies were performed in triple-negative breast cancer (TNBC) models, as the phase I trial is planned for patients with TNBC. Biological functions of virus-encoded transgenes were confirmed, and the virus demonstrated anti-tumor efficacy against TNBC models in mice. In a good laboratory practice (GLP) toxicology study, the virus did not produce any observable adverse effects in mice, suggesting that the doses proposed for the clinical trial should be well tolerated in patients. Furthermore, no neurotoxic effects in mice were seen following intracranial injection of the virus. Also, the risk for horizontal transmission of CF33-hNIS-anti-PD-L1 was assessed in mice, and our results suggest that the virus is unlikely to transmit from infected patients to healthy individuals. Finally, the in-use stability and compatibility of CF33-hNIS-anti-PD-L1 tested under different conditions mimicking the clinical scenarios confirmed the suitability of the virus in clinical settings. The results of these preclinical studies support the use of CF33-hNIS-anti-PD-L1 in a first-in-human trial in patients with TNBC.

INTRODUCTION

Oncolytic viruses are designed to specifically replicate in cancer cells and kill them while leaving normal cells unharmed. A wide variety of viruses, either modified or in their natural form, have been studied for their oncolytic potentials. Many oncolytic viruses have been studied in clinical trials for the treatment of different types of malignancies.[1] Thus far, only one oncolytic virus, talimogene laherparepvec (T-Vec), has received U.S. Food and Drug Administration (FDA) approval. T-Vec, which is approved for the treatment of metastatic melanoma, is an engineered herpes simplex virus 1 (HSV-1) that is armed with the cytokine granulocyte macrophage colony-stimulating factor (GM-CSF).[2] The approval of T-Vec has resulted in a surge of interest in the field of oncolytic virotherapy.

In addition to direct cell lysis, oncolytic viruses also rely on activation of anti-tumor immunity for their efficacy.[3,4] It is therefore not surprising that oncolytic viruses show better efficacy when combined with other therapeutics that are known to avert immune suppression, such as the immune checkpoint inhibitors (ICIs).[4] Backed by robust preclinical data, many clinical trials are ongoing in which oncolytic viruses are being tested in combination with checkpoint inhibitors.[1] Although most of these studies are still ongoing, results available from a few completed studies show that the combination of oncolytic virus and ICIs works well in patients. In phase I clinical trials, T-Vec with ipilimumab (a cytotoxic T lymphocyte-associated antigen 4 [CTLA-4] monoclonal antibody [mAb]) or pembrolizumab (a programmed cell death protein 1 [PD-1] mAb) has shown excellent efficacy in melanoma patients.[5,6] Consequently, several ongoing phase II trials are evaluating T-Vec in combination with ICIs for the treatment of different types of malignancies.[1]

Currently, T-Vec and ICIs cost well over $100,000 each for one course of treatment.[7,8] Given the high costs of ICIs, it will be beneficial to arm oncolytic viruses with transgenes encoding for ICIs. With this in mind, we have engineered a poxvirus, CF33, to encode a single-chain variable fragment (scFv) against programmed death ligand-1 (PD-L1). Furthermore, we have also armed this virus with human sodium iodide symporter (hNIS) gene, which would allow tracking of the

Received 16 September 2021; accepted 4 December 2021;
https://doi.org/10.1016/j.omtm.2021.12.002.

Correspondence: Shyambabu Chaurasiya, PhD, Department of Surgery, City of Hope National Medical Center, Familian Science building, Room#1100 1500 E Duarte Road, Duarte, CA 91010, USA.
E-mail: schaurasiya@coh.org

 © 2021 The Authors.
This is an open access article under the CC BY license (http://creativecommons.org/licenses/by/4.0/).

Table 1. Genome size and percentage identity of CF33 genome with those of parental viruses

Viruses	Genome Size, bp	Identities of CF33 to Genome, %	CF33 Coverage, %
CF33	189,415	100	100
VACV CL	184,042	99.24	95
VACV LC	190,579	99.00	97
VACV AS	177,923	99.19	88
VACV WR	194,711	99.38	97
VACV IHDW1	195,821	99.12	99
VACV Lister	187,893	99.22	96
Cowpox	224,499	97.54	99
Rabbitpox	197,731	99.32	99
Raccoonpox	214,699	87.04	83

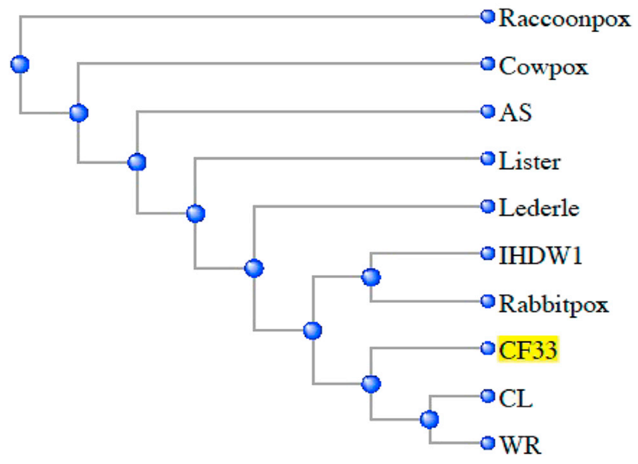

Figure 1. Phylogenetic tree of ten viruses created using the National Center for Biotechnology Information's (NCBI) multiple sequence alignment tool

virus *in vivo* using positron emission tomographic (PET) imaging.[9] To support the testing of this doubly armed oncolytic virus, CF33-hNIS-anti-PD-L1 (also called HOV-3), in a phase I trial in patients with triple-negative breast cancer (TNBC), we have performed preclinical pharmacology studies encompassing primary and secondary pharmacodynamics and biodistribution and safety studies.

RESULTS

Sequence analysis of CF33

CF33 is a chimeric poxvirus generated through homologous recombination among nine strains/species of poxviruses.[10] Simple alignment of CF33 genome sequence with the nine parental viruses shows that the Western Reserve (WR) strain of vaccinia virus (VACV) has the highest similarity (99.38%), while raccoonpox has the lowest similarity (87.04%) to CF33 (Table 1; Figure 1). Also, rabbitpox, IHD, and cowpox demonstrate the highest coverage (>99%) compared with the other six viruses (Table 1). However, it is hard to determine the origins of the CF33 genome regions from simple alignment. Therefore, we applied Kalign[11] to CF33 and the nine parental viruses for multiple sequence alignments. Previously, we had performed the sequence analysis of CF33 using only seven parental viruses whose sequences were publicly available.[10] However, we recently performed sequencing of the remaining two parental viruses (VACV CL and VACV LC), and here we report the results of re-analysis of CF33 genome sequence compared against all nine parental viruses. The identities were calculated and viewed in SnapGene (Figure 2). The whole CF33 genome (189,415 bp) can be resolved into 41 segments, ranging from 353 to 40,691 bp. These segments were found to be derived mainly from three VACV strains: IHD, Lister, and WR, accounting for 60% of the whole CF33 genome. No sequence from raccoonpox or cowpox was detected in CF33 genome.

Poxvirus genes known to directly or indirectly play a role in attachment, entry, replication, and pathogenesis of virus were identified from the literature, and those genes in CF33 were mapped to the nine parental viruses. Our data show diverse parental origin of those genes (Table S1). Ten of 15 pathogenesis-related genes (C21L, C21L, A46R, A52R, SPI-2/CrmA, B18R, C12L, N1L, B8R, and E3L)[12] originated from the IHD strain of VACV. Interestingly, the entry genes came mainly from Lister and WR (10 of 11), such as I2L and J5L from Lister and G9R and L1R from WR. Moreover, genes involved in DNA replication, cell-cell fusion, and attachment were found to originate mainly from IHD and WR, such as A50R (DNA ligase) and D5R (helicase-primase), which originated from IHD and WR, respectively. Furthermore, genes involved in nucleotide metabolism (F4L, A48R, and J2R) are derived from the IHD strain.

In-use stability and device compatibility of CF33-hNIS-anti-PD-L1

We performed studies to determine stability of the virus at room temperature and on ice after thawing. We also determined the stability of virus after multiple freeze-thaw cycles. Furthermore, we determined the stability of the virus in a delivery device (i.e., syringe) for up to 2 h. The virus shows excellent stability at all the conditions tested except in the freeze-thaw cycle study with the lower concentration (1E6 plaque-forming units [PFU]/mL) of the virus (Figures 3A–3C). It was surprising that the virus at high concentration (1E08 PFU/mL) showed no change in titer for up to five freeze-thaw cycles, whereas the same virus at lower concentration (1E06 PFU/mL) showed drastic reduction in titer after every freeze-thaw cycle (Figure 3B). The reason for this discrepancy is not known.

In order to determine the genome stability of the virus during passaging, we sequenced virus genome after every round of passaging for up to five passages (P). Of the five samples (P1–P5), DNA from P2 sample was somehow degraded, and hence sequencing was not performed on the P2 sample. PacBio sequencing was performed on P1, P3, P4, and P5 samples. For analysis, sequence of P1 was used as reference sequence to compare sequences from P3, P4, and P5 to determine the extent of mutations in those late passages of virus. Results of the analysis are shown in Table 2. The comparison between P1

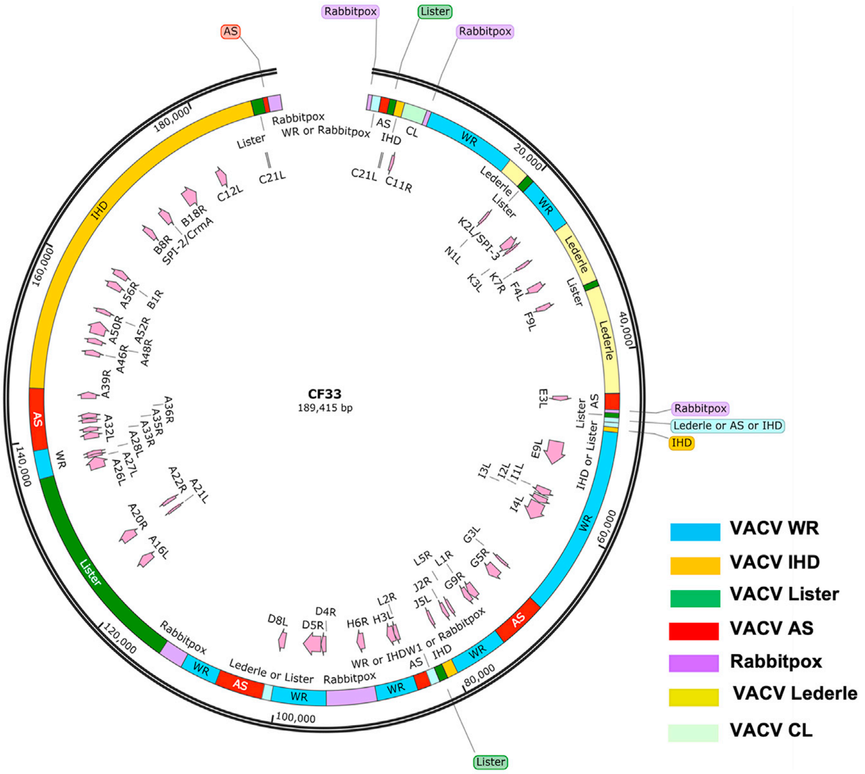

Figure 2. Genomic analysis of CF33

Map of virus genome showing components of parental viruses. Genomic regions are colored according to their parental viruses. Also shown are the origin of genes involved in attachment (A26L, H3L, D8L, and A27L), entry (A21L, G3L, H2R, A28L, F9L, L5R, I2L, J5L, A16L, G9R, and L1R), nucleotide metabolism (F4L, I4L, J2R, and A48R), DNA replication (G5R, E9L, I1L, A20R, A32L, A50R, B1R, D4R, H6R, I3L, D5R, and A22R), prevention of super-infection (A33R and A36R), cell-cell fusion (A56R and K2L), and pathogenesis (A39R, A35R,C21L, A46R, A52R,CrmA, E3L, K3L, K7R, N1L, C11R, B8R, and B18R).

(Seed Stock) and its passages (P3, P4, and P5) indicated two variances (locations 437 and 438) shared among the three passages (Table 2). These two variants occurred within or near homopolymer regions, and it has been reported that deletion sequencing errors are very common in homopolymer regions with PacBio sequencing.[13] However, these two variants shared among three samples tend to be real. Therefore, to determine if the two variants may alter gene expression, P1 assembly was annotated using VGAS, resulting in 272 genes ranging from 126 to 3,495 bp. Sequence of these genes were aligned against P3, P4, and P5 assemblies using Blast. The results show that these genes are conserved (100% sequence similarity) among the four passages (data not shown). Hence, the two variants are not likely to cause any alteration in the biological function of gene products.

Replication of CF33-hNIS-anti-PD-L1 is attenuated in normal cells

We compared the replication kinetics of the virus with that of parental strain (CF33) as well as with the three wild-type strains (IHD, Lister, and WR), which constitute the majority of the CF33 genome. Compared with CF33, replication of CF33-hNIS-anti-PD-L1 virus was found to be about 1 log lower in the human normal cell lines HDFa and Hs27 at all three time points tested (Figure 4). Also, replication of CF33-hNIS-anti-PD-L1 was lower than the wild-type viruses (Lister, IHD, and WR) in both human cell lines except for the Lister strain in Hs27 cells. The Lister strain of VACV seems to have reduced replication in Hs27 cells, to a level similar to CF33-hNIS-anti-PD-L1. In the murine normal cell line EpH4-EV, both CF33 and CF33-hNIS-anti-PD-L1 have lower replication compared with the WR and IHD strains of VACV. Again, the Lister strain has reduced replication in this cell line. Finally, CF33-hNIS-anti-PD-L1 was found to have no replication in NIH3T3 cells at any time point (Figure 4).

Toxicology and biodistribution study

A GLP toxicology study was performed by a third party (Comparative Biosciences, Inc) to determine the toxicity and biodistribution of CF33-hNIS-anti-PD-L1 in an orthotopic syngeneic murine model using C57Bl/6 mice implanted with E0771 cells. The objectives of the study were to assess (1) toxicity and biodistribution of a single intratumorally (i.t.) administered CF33-hNIS-anti-PD-L1 in tumor-bearing mice in 3 weeks and (2) toxicity and biodistribution of repeated intratumorally administered CF33-hNIS-anti-PD-L1 in tumor-bearing mice in 12 days and after a 9 day recovery. The study consisted of 25 groups of C57BL/6 mice: groups 1–7 for toxicology (histopathology), groups 8–14 for tissue biodistribution, groups 15–19 for serum troponin assays, and groups 20–25 for serum cytokine assays (Tables S2–S5). Virus at 1E03, 1E04, or 1E05 PFU was used for single or repeat injections. The highest dose (1E05 PFU) in mice would translate to approximately 5E08 PFU. These doses were chosen to allow testing of virus doses ranging from 1E06 to 5E08 PFUs in humans.

In the 3 week study period, no virus-related abnormalities were found in daily clinical observations or weekly body condition scores. Furthermore, once- or twice-weekly measurements of body weight, food consumption, and tumor size showed no statistically significant differences in males or females among the single-dose groups (groups 1–4) or among the repeat-dose groups (groups 5–7) (data not shown).

Blood levels of cytokines, IFN-g, IL-1b, IL-6, IL-10, TNF-a, and TGF-β1, were tested at 3 h, day 7, and day 14 in the single-dose groups and at days 14, 19, and 21 in the repeat-dose groups. There were no

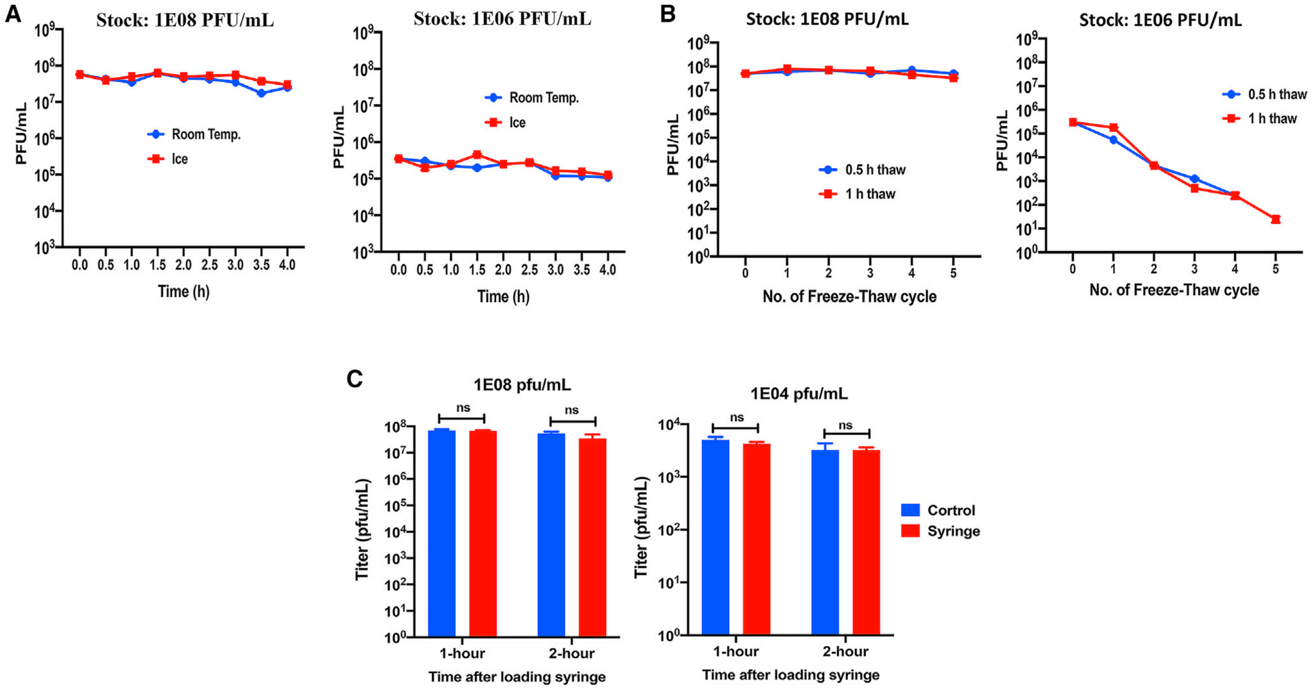

Figure 3. Stability of CF33-hNIS-anti-PD-L1

(A) CF33-hNIS-anti-PD-L1 at 1E08 or 1E06 PFU/mL was thawed and placed either at room temperature (RT) or on ice. Aliquots from all vials were taken immediately after thawing (0 h) and at 0.5, 1, 1.5, 2, 2.5, 3, 3.5, and 4 h. At each time point, aliquots of virus taken from the vials were immediately used to infect CV-1 cells for titration. Mean ± SD is plotted. (B) Stability of CF33-hNIS-anti-PD-L1 after multiple freeze-thaw cycles. Virus stocks at 1E08 or 1E06 PFU/mL were freeze-thawed for up to five cycles (frozen for 1 h at −80°C and thawed for 30 min or 1 h at RT). After every thaw, aliquots were taken and immediately used to infect CV-1 cells for titration. Mean ± SD is plotted. (C) Stability of the virus in syringe. Viruses at 1E08 or 1e04 PFU/mL were loaded in 10 mL syringes fitted with 25G needles and incubated for 1 or 2 h at RT. At the end of 1 and 2 h incubation, virus was ejected from the syringes and immediately used to infect CV-1 cells for titration. Virus incubated at room temperature in an Eppendorf tube was used as control.

changes in the cytokine levels compared with vehicle controls except that TGF-β1 levels were significantly higher in the repeat-high-dose group at all time points compared with the vehicle control group (Tables S6–S10). Cardiac troponin I levels were below the limit of quantification (<7.82 pg/mL) in all samples.

For histopathological examinations, mice were euthanized at 24 h, 7 days, and 21 days after treatment for single-dose groups (groups 1–4) and at 12 and 21 days after first injection for repeat-dose groups (groups 5–7). Tissues included in the histopathological study were : adrenal gland, artery, aorta, femur, bone marrow, sternum, brain, esophagus, eye. heart:, intestines, cecum, colon, duodenum, ileum, jejunum, kidney. liver with gall bladder, lung, lymph node, mandibular and mesentric lymph nodes, muscle, hindlimb, sciatic nerve, oral tissues, tongue, ovary with oviduct, pancreas, salivary gland, skin, spinal cord, spinal cord with cervical portion, spinal cord with lumbar portion, thorax, spleen, stomach, thymus, thyroid with parathyroid, trachea, tumor, urinary bladder, uterus, and vagina. From histopathology examination, no virus-related microscopic findings were detected in any of the treatment groups, and the pathologist concluded that CF33-hNIS-anti-PD-L1 was well tolerated at all doses tested.

Biodistribution of virus was assessed using two techniques: (1) standard plaque assay and (2) quantitative PCR (qPCR) in groups 8–14 (Table 3). Tissues and samples used for biodistribution studies were: blood, urine, feces, saliva, bladder, brain, heart, intestine, kidney, liver, lung, muscle, ovaries, spleen, and tumors.

Among the 15 tissue and sample types tested by qPCR, the only sample showing the presence of viral DNA was tumor. Specifically, viral DNA was detectable in two of five tumor samples at day 7 and 0 of 5 samples at days 19 and 21 in the single-high-dose group. In the repeat-high-dose group, viral DNA was also detectable in 3 of 5 tumor samples at day 12 and 1 of 5 samples at days 19 and 21. None of the tumor samples in the vehicle control groups had detectable viral DNA. Data from the qPCR analysis for all organs are included in Table S11.

Among the 15 tissue and sample types tested in the standard plaque assay, infectious virus was detectable only in the tumor samples of the single-high-dose group (1 of 5; 500 PFU/g) at day 7 and repeat-high-dose group (2 of 5; 100 and 455 PFU/g) at day 12. The tumor samples at the later time points of these two groups and all the tumor samples in the vehicle control groups were found to be negative

Table 2. Detected variants from *de novo* assemblies (HOV-3 P3, P4, and P5)

Reference	Sample	Location	Type	Mismatched Base	Context
HOV-3 P1 (191,820 bp)	P3	437	substitution	T → A	AAATTT
		438	substitution	A → T	AAATTT
	P4	437	substitution	T → A	AAATTT
		438	substitution	A → T	AAATTT
	P5	437	substitution	T → A	AAATTT
		438	substitution	A → T	AAATTT

for infectious virus. The qPCR and plaque assay results suggest that after administration, the virus was specifically distributed in the administered tumors, and the levels in tumors decreased over time.

Assessment of neurotoxic effect of the virus

Wild-type vaccinia virus such as the WR strain has been shown to cause severe neurotoxicity in mice at a dose as low as 100 PFU[14,15] when injected intracranially. In order to rule out the possibility of a neurotoxic effect of CF33-hNIS-anti-PD-L1, we used CF33-hNIS-anti-PD-L1 virus at a dose 10 times higher (1,000 PFU) than the dose reported to cause severe neurotoxicity for wild-type vaccinia virus. C57BL/6 mice of both genders were injected intracranially with 1,000 PFU CF33-hNIS-anti-PD-L1 virus or PBS as control. Mice were closely monitored for 30 days for signs of toxicity, and their body weights were measured daily. At the end of the observation period, mice were euthanized, and histopathological analysis was performed on their brains by a veterinary pathologist. No overt sign of toxicity was observed in the mice, and change in weight of the mice in virus-injected group was very similar to that for PBS-injected group (Figure 5). Furthermore, analysis of brain sections showed no virus-related adverse effects at the injection site. Also, away from the cerebellar injection region, brain sections of virus-treated animals looked very similar to those of the PBS-treated animals, suggesting that there was no virus replication in brain cells and hence no killing of brain cells by the virus. Taken together, these data show that CF33-hNIS-anti-PD-L1 does not have any clinically meaningful neurotoxic effect in mice at the dose tested.

Assessment of risk for horizontal transmission

The risk for horizontal transmission was also assessed for CF33-hNIS-anti-PD-L1 virus. To assess the risk, we injected E0771 tumor-bearing mice intratumorally with 1E06 PFU CF33-hNIS-anti-PD-L1 and then housed them with other mice, with or without tumors, that were not injected with virus, as shown in Figure 6A. The dose of virus used in this study was chosen at 10-fold higher than the highest dose used in the toxicology study, to rule out the possibility of virus transmission from injected to non-injected mice. To mimic the clinical situation in which patients receiving CF33-hNIS-anti-PD-L1 would be isolated for at least 24 h, we isolated virus-injected mice for 24 h in a separate cage. Following the 24 h isolation, virus-injected and non-injected mice were housed together such that each cage had at least one mouse that had been injected with virus. Finally, virus titers were measured in the tumors and normal tissues of all mice from a cage at day 7, day 14, and day 21, to determine whether the virus could be detected from non-injected mice. Our results indicate that the CF33-hNIS-anti-PD-L1 virus did not transmit from injected to non-injected mice, as no virus was detected in tumor or tissues of non-injected mice. Also, even in the injected mice, virus was detectable only in the tumors. Furthermore, no virus was detected even in injected tumor at 3 weeks post-injection (Figure 6B), suggesting that virus may have been completely cleared by the immune system at this time point.

Transgenes in CF33-hNIS-anti-PD-L1 make functional proteins

The anti-PD-L1 cDNA in the virus is composed of an Igκ light chain leader sequence, the VH chain sequence of an anti-PD-L1 monoclonal antibody, a (G4S)3 linker sequence, the VL chain sequence, and a C-terminal FLAG tag (DDDDK tag). The anti-PD-L1 expression cassette is under the control of the vaccinia H5 promoter. First, we used Western blot analysis to detect the expression of anti-PD-L1 from virus-infected breast cancer cells (Figure 7A). We then evaluated the ability of virus-encoded anti-PD-L1 to block PD-L1 on the surface of human and murine breast cancer cells. Mouse breast cancer cells, E0771, were pre-incubated with purified virus-encoded anti-PD-L1 or control antibody (atezolizumab), followed by staining with anti-mouse PD-L1-PE antibody. Cells were then analyzed using flow cytometry. Our results demonstrate that virus-encoded anti-PD-L1 scFv blocks the detection antibody from binding to PD-L1 on mouse breast cancer cells E0771, in a dose-dependent manner (Figure 7B). Similar results were found against the human breast cancer cell line MDA-MB-468 (data not shown).

Expression of the transgene hNIS and localization of the protein on cell surface was confirmed using immunofluorescent staining of hNIS *in vitro* (Figure 7C). Immunofluorescence staining was performed 24 h after infection of cells at a multiplicity of infection (MOI) of 0.01.

CF33-hNIS-anti-PD-L1 favorably modulates immune cells and exhibits anti-tumor efficacy in the murine syngeneic model E0771

Mice bearing E0771 tumors were injected with a single intratumoral injection of 1E05, 1E06, or 1E07 PFU CF33-hNIS-anti-PD-L1 or PBS and euthanized 7 days later. Tumor volume and weight of mice were measured before treatment and immediately before euthanasia. No

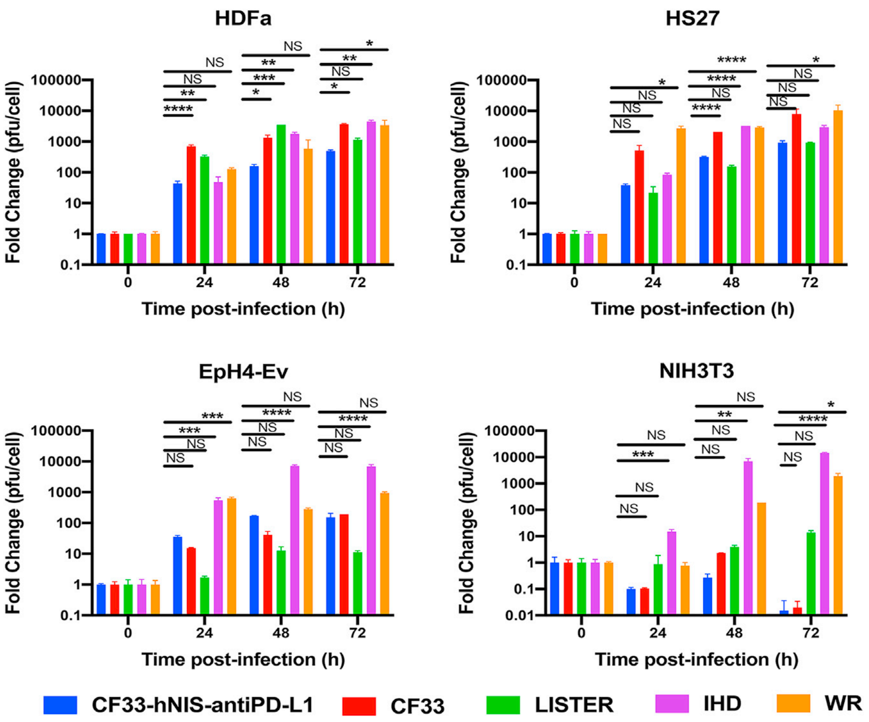

Figure 4. Replication of viruses in normal cells

Normal cell lines of human (HDFa and HS27) or murine (EpH4-Ev and NIH3T3) origin were infected with the indicated viruses at an MOI of 0.01, and virus titers were determined at indicated time points. Fold change in PFU/cell was calculated by dividing the virus titers at different time points with the titer of virus used for infection. Mean ± SD is plotted.

significant difference was observed between the average weight of mice treated with PBS and any of the virus doses (Figure 8A). Average tumor volume for virus-treated groups were less than the PBS-treated group (Figure 8B) over the 1 week time period, but because of high variation within the PBS group the difference did not reach statistical significance.

Tumors harvested 1 week post-treatment were divided into two parts; one part was used for immune cell analysis using flow cytometry and the other part for quantification of anti-PD-L1 and CF33-hNIS-anti-PD-L1 virus using qPCR. Interestingly, although there was a trend toward a higher amount of viral DNA in tumors treated with higher doses of virus, differences between the highest and lowest dose groups were not statistically significant (Figure 8C). As for the levels of anti-PD-L1, four of six mice in the lowest dose group (1E05 PFU), six of six mice in the mid-dose group (1E06 PFU), and five of six mice in the highest dose group (1E07 PFU) had detectable levels of anti-PD-L1. Although there was no significant difference in the levels of anti-PD-L1 between low and mid doses, the highest dose group had significantly higher levels of anti-PD-L1 (Figure 8D). The fact that anti-PD-L1 level was markedly different in mice treated with the highest dose suggests that the dose of virus needs to reach or exceed a certain threshold (1E07 PFUs in this case) to produce an abundant amount of product from their transgenes.

Immune cells were analyzed in tumor, blood and spleen and levels of different immune subsets were compared among the treatment groups. Levels of immune cells (CD45+) in the tumors treated with the highest dose (1E07 PFU) of the CF33-hNIS-anti-PD-L1 were higher than those in PBS-treated tumors or tumors treated with lower doses of virus (Figure 9). However, because of large variation within treatment groups, the difference did not attain statistical significance. Interestingly, levels of CD8+ T cells were significantly higher in the tumors of the mid- and high-dose groups compared with the PBS group. Among the virus-treated groups, levels of CD8+ T cells seem to correlate with the doses of virus (Figure 9). Similar results were observed for blood and spleen (data not shown). Levels of PD-L1 in non-immune cells (CD45− cells, mostly tumor cells) were found to be significantly higher in the low-dose group compared with the PBS group (Figure 9), which is consistent with our previous findings that cancer cells upregulate PD-L1 expression in response to CF33 virus.[16] However, compared with the low-dose group, the mid- and high-dose groups showed lower levels of PD-L1 in non-immune cells. This is probably due to blockade of PD-L1 by virus-encoded anti-PD-L1.

CF33-hNIS-anti-PD-L1 exhibits anti-tumor efficacy against 4T1 tumor model

In this study, we evaluated the safety and anti-tumor efficacy of CF33-hNIS-anti-PD-L1 in the highly aggressive 4T1 model. BALB/c mice bearing bilateral orthotopic tumors were injected with virus or PBS on days 1, 3, and 5 either intratumorally or intravenously (i.v.). Virus was injected (1E07 PFU) to each tumor, or 2E07 PFU was injected per mouse intravenously. The virus treatment was well tolerated in mice, as evidenced by weight change (Figure 10A). Both the i.t. and i.v. treatment with CF33-hNIS-anti-PD-L1 demonstrated some levels of anti-tumor efficacy and significantly increased survival of the virus-treated mice (Figures 10B and 10C).

CF33-hNIS-anti-PD-L1 shows excellent anti-tumor efficacy against a TNBC xenograft model

In order to test the efficacy of the virus against TNBC of human origin, we used a xenograft model in which tumors were generated in athymic mice using human TNBC cells MDA-MB-468. Mice were treated intratumorally with a single injection of CF33-hNIS-anti-PD-L1 at doses that were used in the toxicology study (1E03, 1E04, or 1E05 PFU) and were monitored for weight change as a surrogate of toxicity, and tumor regression for anti-tumor efficacy. In this study, mice treated

Table 3. Groups of mice used in biodistribution study

Group	Number/Sex of Mice	Animal Number (F)	Tumor Induction	Test Article Treatment		In-Life Procedure	Termination	Terminal Procedure
8	5/F	851–855	Yes	i.t. 1× day 0	control article (vehicle)	clinical observations: 1× daily body condition scoring: weekly body weights: pre-study and necropsy	day 7	terminal blood and tissue collection for viral plaques and PCR
	8/F	856–863					day 21	
9	5/F	951–955			CF33-hNIS-anti-PD-L1 1E03 PFU		day 7	
	8/F	956–963					day 21	
10	5/F	1051–1055			CF33-hNIS-anti-PD-L1 1E04 PFU		day 7	
	8/F	1056–1063					day 21	
11	5/F	1151–1155			CF33-hNIS-anti-PD-L1 1E05 PFU		day 7	
	8/F	1156–1163					day 21	
12	5/F	1251–1255		i.t. 6× days 0–12	control article (vehicle)		day 12	
	8/F	1256–1263					day 21	
13	5/F	1351–1355			CF33-hNIS-anti-PD-L1 1E03 PFU		day 12	
	8/F	1356–1363					day 21	
14	5/F	1451–1455			CF33-hNIS-anti-PD-L1 1E05 PFU		day 12	
	8/F	1456–1463					day 21	

The table shows only female mice. The same number of male mice were also treated in the exact same manner.

with different doses of virus gained weight similarly to mice treated with the control (PBS) (Figure 11A). This suggests that all doses of CF33-hNIS-anti-PD-L1 used in this study were well tolerated. Furthermore, all three doses of CF33-hNIS-anti-PD-L1 virus were able to inhibit growth of the tumor (Figure 11B), indicating a robust anti-tumor potency of this virus against TNBC of human origin.

DISCUSSION

The objectives of these studies were to evaluate the risks and benefits of using the oncolytic virus CF33-hNIS-anti-PD-L1 to support clinical development in patients with TNBC. Because CF33 is a chimeric poxvirus generated from multiple species and strains of poxviruses, it was important to perform a thorough investigation of the viral genome and its stability. We compared the genome of CF33 with the genome of all nine parental viruses and determined the origin of sequences in CF33. The majority of the CF33 genome is derived from the vaccinia virus strains IHD, Lister, and WR, whereas no sequence from raccoonpox or cowpox was detected. Also, in CF33 most of the genes involved in viral DNA replication and pathogenesis are derived from the VACV strain IHD, which was used as a vaccine against smallpox.

Through a variety of experimental methods, we evaluated the safety associated with the use of the oncolytic virus. First, *in vitro* studies were performed to compare the growth of CF33-hNIS-anti-PD-L1 with CF33 and the parental viruses that make up the majority of CF33 genome (WR, Lister, and IHD) in normal cell lines of human and murine origins. CF33-hNIS-anti-PD-L1 showed attenuated replication compared with CF33 and other wild-type viruses, with the exception of the Lister strain. A GLP toxicology study performed by a third-party company to determine the safety and biodistribution of CF33-hNIS-anti-PD-L1 in tumor bearing C57BL/6 mice treated with single or multiple injections of the virus at various dose levels showed no virus-related toxicity. Furthermore, intracranial injection of the virus at a dose 10 times higher than the lethal dose of WR strain[14,15] in C57Bl/6 mice did not show clinically meaningful virus-related toxicities. Finally, the risk for horizontal transmission of the virus using an experimental setup to mimic the clinical scenario suggested that the risk for horizontal transmission for this virus is minimal.

We evaluated the functionality of the virus-encoded transgene, anti-PD-L1, to confirm that the protein is biologically functional. Functionality of virus-encoded anti-PD-L1 was assessed using an *in vitro* competitive binding assay. Anti-PD-L1 protein purified from virus-infected cells showed a dose-dependent response in the assay, confirming the ability of virus-encoded protein to bind to PD-L1 on cell surface. However, compared with atezolizumab (an FDA-approved anti-PD-L1 mAb) virus-encoded anti-PD-L1 was found to be less effective. This could be partly because the virus-encoded antibody used in this study was not as pure as the atezolizumab. Nevertheless, this assay confirmed that the virus-encoded protein is functional.

Finally, we evaluated anti-tumor efficacy of the virus using xenograft and syngeneic models of TNBC in mice. It should be noted that higher doses of virus were used in syngeneic tumor models for efficacy studies than the doses used in toxicology studies, because murine tumors are less susceptible to the virus, but therapeutic doses in clinical trials will be based on the doses used in toxicology studies. In the E0771 tumor model, immune cell analysis 7 days after virus injection showed a

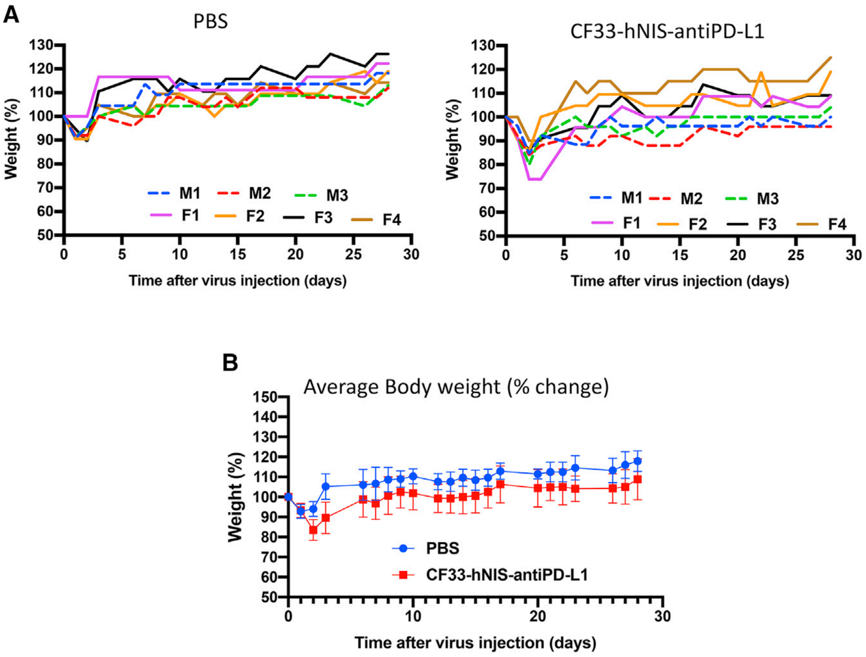

Figure 5. Assessment of neurotoxic effect of CF33-hNIS-anti-PD-L1

Male (M) and female (F) mice were treated intracranially with PBS or 1,000 PFU CF33-hNIS-anti-PD-L1, and their weight was measured every day and plotted. (A) weight of individual mouse in each treatment group is shown. (B) Average weight for each treatment group was plotted and compared. The difference between the two group is not statistically significant.

significantly larger number of CD8+ T cells in tumors of mice that were treated with virus compared with those treated with PBS. This increase in the number of CD8+ T cells in the tumors was found to be dose dependent, which is in agreement with a recently published study by Flores et al.[17] in which the authors showed that a high dose of an oncolytic myxoma virus (1E06 foci-forming units [FFU]) resulted in a significant increase in CD8+ T cells in a melanoma model, whereas a low dose (1E04 FFU) of the same virus failed to do so. The fact that the virus was able to increase tumor infiltration by CD8+ T cells is indicative of positive immune modulation by the virus. However, despite positive immune modulation, the anti-tumor efficacies observed in these syngeneic models were modest. This was not surprising as we have previously shown that murine cancer cells are less susceptible to CF33 compared with human cancer cells.[10] Furthermore, the two syngeneic models used in this study (4T1 and E0771) are extremely aggressive models that are difficult to control. Importantly, in a xenograft model of TNBC (MDA-MB-468), CF33-hNIS-anti-PD-L1 was able to completely control tumor growth at a dose as low as 10^3 PFU, a dose 100- to 1,000-fold lower than those reported for other oncolytic viruses.[18–20] This shows the excellent efficacy of the virus against tumor of human origin.

Taken together, our data show that CF33-hNIS-anti-PD-L1 is a safe and low-risk virus with potent anti-tumor efficacy against TNBC of human origin, and thus these data support further evaluation of the virus in clinical settings.

MATERIALS AND METHODS

Virus

CF33 is a chimeric poxvirus generated through homologous recombination among nine strains/species of poxviruses. Construction of CF33 and CF33-hNIS-anti-PD-L1 has been described elsewhere.[10,21] CF33-hNIS-anti-PD-L1 virus was generated by replacing the viral genes *J2R* and *F14.5L* with hNIS and anti-PD-L1 expression cassettes, respectively.

The CF33-hNIS-anti-PD-L1 clinical lots were manufactured under current good manufacturing practice (CGMP) conditions by infection of A549 cells from an established working cell bank (WCB) with CF33-hNIS and formulated in final formulation buffer consisting of 140 mM NaCl and 10 mM Tris-HCl (pH 7.8) in water for injection (WFI) tested to U.S. Pharmacopeia (USP) specifications. The clinical lots were also assessed for the presence of adventitious virus, mycoplasma, and levels of proteins and nucleic acids as well as endotoxin, and met release test specifications. Clinical-grade virus was used in the GLP study (toxicology) and in some of the non-GLP studies (in-use stability and neurotoxicity studies); laboratory stock virus (amplified in CV-1 cells and purified on sucrose gradients) was used for all other studies. Purification of virus on sucrose gradients was performed by following the protocol published from the laboratory of Bernard Moss.[22]

Intracranial injection

All animal studies were conducted under City of Hope Institutional Animal Care and Use Committee approved protocol in compliance with NIH's guideline for the use of laboratory animals. Mice were injected intraperitoneally (i.p.) with ketamine/xylazine cocktail (90–150 mg/kg ketamine, 7.5–16 mg/kg xylazine), eye ointment was applied, and fur on the head was shaved. An incision of 1 cm was made using a sterile scalpel at the mid-line extending from just behind the eyes to the level of ears. Next, a hole was drilled in the skull at 1 mm lateral from the mid-line and 2 mm posterior to the lamboid suture. 1X PBS (catalog number 21-040-CV; Corning) or diluted CF33-hNIS-anti-PD-L1 virus (3 μL per mouse) was injected using a 10 μL Hamilton syringe with a 28G needle (0.5 μL at 3 mm deep from the dura, 0.5 μL at 2.75 mm deep from the dura, 0.5 μL at 2.5 mm deep from the dura, 0.5 μL at 2.35 mm deep from the dura, 0.5 μL at 2.25 mm deep from the dura, and 0.5 μL at 2.15 mm deep from dura). After retracting the needle, bone wax was used to occlude the burr hole, betadine was applied to the surgical area, and the skin was closed with skin glue. Mice were subcutaneously injected with slow-release buprenorphine. Post-surgical

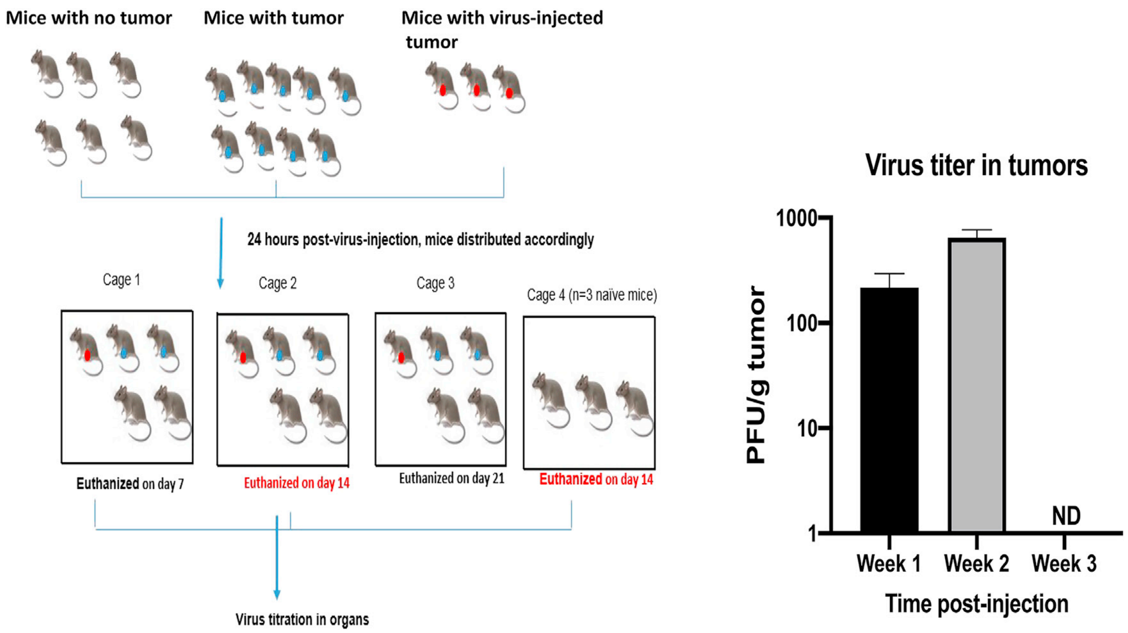

Figure 6. Assessment of risk of horizontal transmission of CF33-hNIS-anti-PD-L1

Left: experimental schema. i.t., intratumoral. Right: titer of CF33-hNIS-anti-PD-L1 in virus-injected tumors. Data represent average ± SD. ND, not detectable.

mice were kept on a heating pad, and "Post-Op" cards were placed on the cages. Mice were monitored and weighed twice a day for the first 10 days, excluding weekends, and then once daily for the next 20 days.

Preparation of brain section

At the end of the study period, mice were euthanized using carbon dioxide (CO_2), and brain as well as other organs (including lung, liver, kidney, testes or ovary, and spleen) were harvested. Immediately after harvesting, brain tissues were placed in 10% neutral-buffered formalin and left at room temperature for 48 h for fixation. After 48 h of fixation, the tissues were sent to the Pathology Core Facility at City of Hope National Medical Center (Duarte, CA) for sectioning and hematoxylin and eosin (H&E) staining. Five sections were obtained for each brain at 5 μM thickness, and sections 3 and 4 from each brain were H&E stained, and slides were shipped to a consultant veterinarian pathologist for assessment of the brain sections.

Virus genome analysis

Genome of CF33 was sequenced using next-generation sequencing. The genomic sequence of CF33 was aligned with nine genome sequences, including VACV strains Ankara (AS; accession number: U94848), Western Reserve (WR; NC_006,998), IHD-W clone IHDW1 (KJ125439), Lister (KX061501), Lederle-Chorioallantoic (LC), Calf-Lymph (CL), cowpox strain Brighton Red (AF482758), rabbitpox strain Utrecht (AY484669), and raccoonpox strain Herman (NC_027,213) using Kalign multiple sequence alignment (version 3).[11] The obtained alignments were processed using the seqinR package (http://seqinr.r-forge.r-project.org/) for the R environment, with trimming the gaps of CF33 sequence in the alignments and identifying the highest identity to any parental strain. If the identities between CF33 and multiple viruses are the same, these regions will be assigned multiple origins. Identity (99.8% or higher) to the parental genomes was confirmed using Basic Local Alignment Search Tool (BLAST). The map of the genome was created using SnapGene Viewer.

Functional gene analysis

Poxvirus genes known to directly or indirectly play roles in attachment, entry, replication, and pathogenesis of virus were identified from the literature,[11,12,23–28] and those genes in CF33 were mapped to the nine parental viruses, indicating diverse parental origins (Table 2). The functional genes in the CF33 genome were also mapped to the parental genomes to determine their origins. If the identities between CF33 and multiple viruses are the same, these regions were assigned multiple origins.

For phylogenetic analysis of viruses, we adopted PTreeRec[29] on the basis of genome BLAST distance to reconstruct the phylogenetic trees of CF33 and the nine parental viruses. The analysis involves three basic steps: (1) regions of maximal segment pairs (MSP) on the basis of an all-against-all pairwise comparison of genomes are located (CF33 and the nine parental genomes were applied), (2) a distance matrix is calculated from MSP scores or coverage, and (3) a phylogenetic tree is reconstructed using the neighbor-joining method.[30]

Virus replication

In order to compare the growth kinetics of the viruses, cells (HS27, HDFa, EpH4-Ev, or NIH3T3) were seeded in 24-well plate at a density of 50,000 cells/well. On the next day, cells were infected with

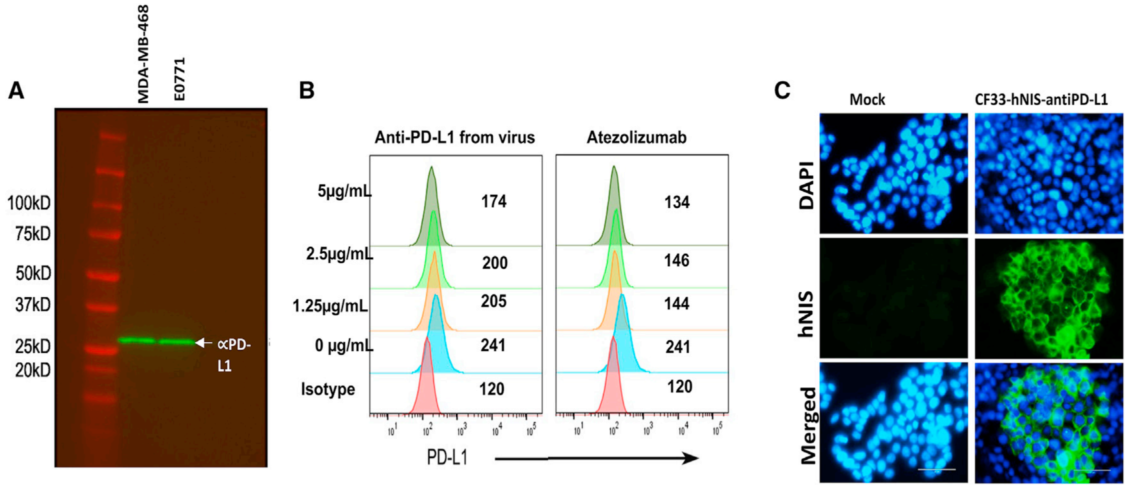

Figure 7. Detection and functionality of virus-encoded transgenes

(A) Detection of anti-PD-L1 in the supernatant of CF33-hNIS-anti-PD-L1-infected human (MDA-MB-468) and murine (E0771) TNBC cells. (B) Virus-encoded anti-PD-L1 antibody blocks surface PD-L1 binding of mouse breast cancer cell line E0771 in a dose-dependent manner. Numbers on the graphs are mean fluorescence intensity (MFI). (C) Immunofluorescent staining of hNIS protein in HT29 cells that were mock-infected (medium only) or infected with CF33-hNIS-anti-PD-L1 virus at an MOI of 0.01.

CF33-hNIS-anti-PD-L1, CF33, VACV Lister, VACV IHD, or VACV WR virus at an MOI of 0.01. After infection, plates were incubated at 37°C. Cell lysates were collected at 24, 48, and 72 h post-infection. Virus titers in the lysates as well as the initial inoculum (0 h) were determined using standard plaque assay on CV-1 cells. Fold change in virus titer (PFU/cell) was calculated relative to amount of virus used for infection (0 h).

Cytotoxicity

To determine cytotoxic potential of the viruses, 3,000 cells from each cell line (HS27, HDFa, EpH4-Ev, or NIH3T3) were seeded per well with 100 μL cell culture medium in 96-well culture plates. The following day, cells were either mock-infected (medium only) or infected with CF33-hNIS-anti-PD-L1, CF33, VACV Lister, VACV IHD, or VACV WR virus at MOIs of 0.1 and 0.01 in duplicate or triplicate. The infected cells were incubated at 37°C for 72 h. Next, CellTiter 96 AQ_{ueous} (Promega) reagent was added to the cells, and 1 h later absorbance was measured at 490 nm using a plate reader (Tecan Spark). Percentage survival was calculated relative to mock-infected wells.

Risk for horizontal transmission

One day after virus injection, mice were re-caged in cage 1, cage 2, or cage 3 (as shown in Figure 6A) such that each cage had one mouse injected intratumorally with virus and four non-injected mice (two tumor-bearing and two non-tumor-bearing). The fourth cage contained three naive mice (i.e., mice with no tumors and no virus injection).

Passaging of virus

Seed stock virus was used to infect the human lung cancer cell line A549 at low MOI (about 0.01 PFU/cell). Briefly, 2 × T225 flasks were seeded with 5E06 cells/flask containing Dulbecco's modified Eagle's medium (DMEM)/F12 media supplemented with 10% fetal bovine serum (FBS). Cells were infected with CF33-hNIS-anti-PD-L1 at an MOI of 0.01 and incubated. The media containing the virus was DMEM supplemented with 2.5% FBS. After 2 h following incubation, DMEM supplemented with 10% FBS was added to the infected cells.

Cell lysates were collected post-infection, virus was purified (passage 1) on sucrose cushion, and DNA was isolated for sequencing. Virus from P1 was used to perform the second round of infection in A549 cells to generate P2 virus, and DNA was isolated from P2 for sequencing. This process was repeated to get CF33-hNIS-anti-PD-L1 virus from up to five passages (Figure 12). At each passage, infected A549 cells were harvested and purified on a sucrose cushion per the manufacturer's protocol. DNA was harvested and sent to the Integrative Genomics Core at City of Hope National Medical Center for sequencing and analysis.

Sequencing and analysis

DNA library preparation and sequencing were performed by the Integrative Genomics Core.

Blockade bioassay of purified anti-PD-L1 antibody

Anti-PD-L1 scFv was purified using Pierce Anti-DYKDDDDK Magnetic Agarose (catalog #A36798; Thermo Fisher Scientific) per the manufacturer's protocol. Blockade bioassay of surface PD-L1 binding was performed using flow cytometry in the presence of an exogenous anti-PD-L1 antibody. Briefly, MDA-MB-468 or EO771 cells were incubated with exogenous anti-PD-L1 antibodies (purified anti-PD-L1 antibodies from virus-infected cells or positive controls: anti-human PD-L1 antibody clone 29E.2A3 or commercial anti-mouse PD-L1 antibody clone10F.9G2 or the FDA-approved atezolizumab) for 20 min on ice. Next, PE-conjugated detection antibodies were added, that is anti-human PD-L1-PE (clone 29E.2A3) for human

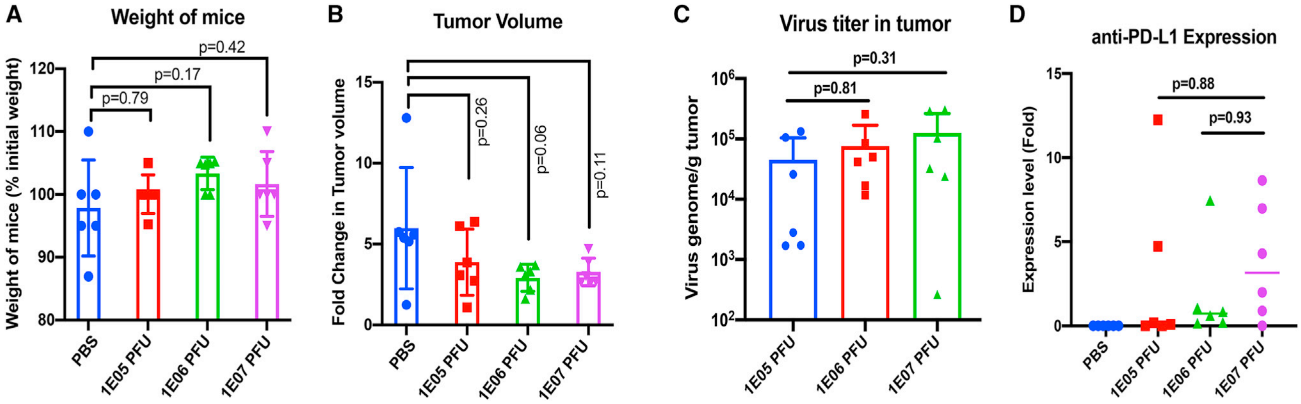

Figure 8. Safety and anti-tumor efficacy in E0771 model

(A) Mice (n = 6/group) were weighed immediately before virus injection and again 7 days later at the time of euthanasia. Weight of mice at the time of euthanasia was determined as percentage of the weight before virus injection. (B) Tumor volume was measured on the day of virus injection and again 7 days later on the day of euthanasia. Tumor volume was calculated using the equation: volume = length × $(\text{breadth})^2/2$. Fold change in tumor volume was calculated by dividing the tumor volume on the day of euthanasia by the volume on the day of virus injection for individual mouse. Mean ± SD is plotted. (C) Copy number of virus genome was determined using qPCR as described in materials and methods. (D) Expression levels of anti-PD-L1 in tumors were determined using qPCR and compared among the groups treated with different doses of virus. ND, not detectable.

breast cancer cells MDA-MB-468 and anti-mouse PD-L1-PE (clone 10F.9G2) for mouse breast cancer cells EO771, and incubated on ice for 20 min. Following incubation, cells were washed with PBS containing 2% fetal bovine serum and fixed with 4% paraformaldehyde. Finally, cells were analyzed using a BD LSRFortessa Flow Cytometer, and FlowJo software was used for data analysis. Results are shown as histograms and mean fluorescence intensity (MFI).

For verification of purified antibody using western blot, purified anti-PD-L1 antibody was loaded onto Criterion Precast Gel and the Criterion Vertical Electrophoresis Cell System (Bio-Rad). Following electrophoresis, the gel was transferred to a polyvinylidene fluoride (PVDF) membrane and stained with rabbit anti-FLAG primary antibody (catalog #GTX77457; GeneTex) followed by anti-rabbit secondary antibody conjugated with the IRDye 800C (Li-CoR) and imaged using the Azure Biosystems C600 Image System. Anti-PD-L1 single-chain antibody was visualized at 26 kD (Figure 2).

Tumor models

Human TNBC cells (MDA-MB-468) were cultured in RPMI-1640 and tested for mycoplasma using the MycoAlert Mycoplasma Detection Kit as per the manufacturer's protocol (Lonza). MDA-MB-468 cells were harvested at approximately 80% confluency, using trypsin, and washed twice with 1X PBS. These cells were then counted and prepared in one part Matrigel to one part PBS at 5E06 cells/50 μL. Athymic (nude) mice were anesthetized using isoflurane, and sterile eye ointment was applied to their eyes to avoid dryness. A 24G needle was used to inject 50 μL cells into the abdominal mammary fat pad of each mouse within 3 h of preparation.

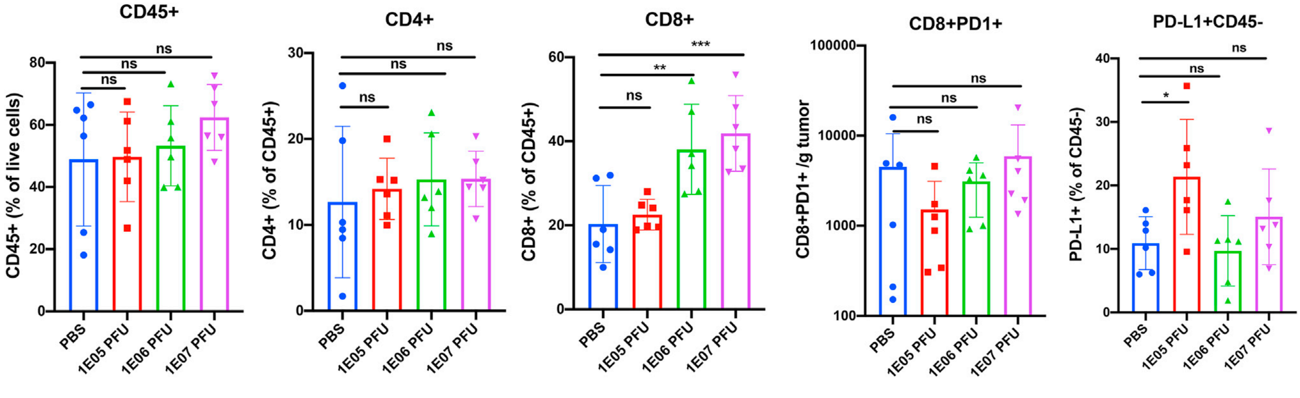

Figure 9. Immune cell analysis in E0771 model

Mice bearing E0771 tumors were injected intra-tumotally with PBS or indicated doses of the virus. One week after treatment, tumors were harvested, and immune cells were quantified using flow cytometry. Comparison of the proportion of different immune cell subsets in the tumors of mice treated with PBS or indicated doses of CF33-hNIS-anti-PD-L1. *p < 0.05, **p < 0.01, and ***p < 0.005, one-way ANOVA; NS, not significant.

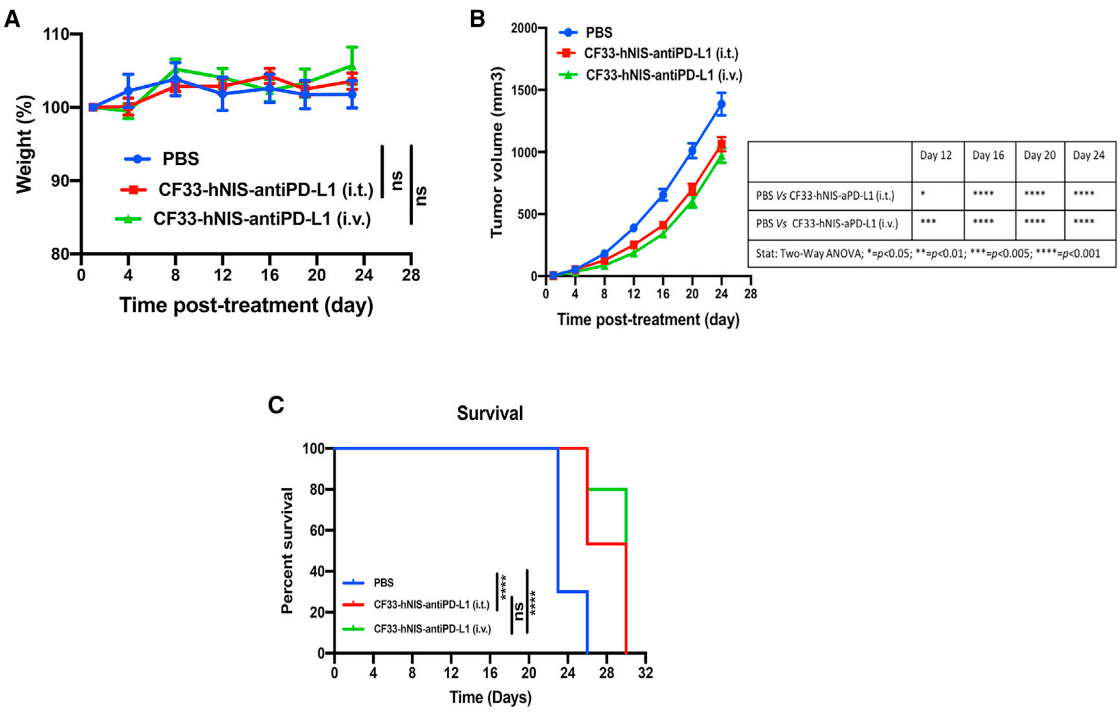

Figure 10. Safety and anti-tumor efficacy in 4T1 model

4T1 tumor-bearing mice (n = 10 or n = 15 mice/group; each mouse has 2 tumors, i.e., n = 20 or n = 30 tumors/group) were treated with PBS or virus. (A) Weight of mice was measured and compared among the treatment groups. (B) Tumors were measured twice weekly, and tumor volume was calculated using the equation: volume = length $\times$ (breadth)^2/2. Average volume $\pm$ SEM is plotted. Statistical analysis was performed using two-way ANOVA with Dunnett's multiple-comparisons test. (C) Mice were euthanized when tumor volume reached the maximum allowed tumor burden (1,500 mm^3). **** = $p < 0.0001$, log rank (Mantel-Cox) test. NS, not significant.

Murine TNBC cells E0771 were cultured in RPMI-1640 and prepared as described above. 1E05 E0771 cells were injected in anesthetized C57BL/6 mice within 3 h. Similarly, 4T1 cells were prepared in one part Matrigel to one part PBS at a concentration of 5E04 cells/50 μL. 4T1 cells (50 μL [5E04]) were injected subcutaneously into the abdominal mammary fat pad on both sides to generate bilateral tumors in BALB/c mice.

For all tumor models, virus treatment was initiated when the tumor volumes were in the range of 50–100 mm^3.

Flow cytometry

Single cells were generated from tumors and spleens using the gentleMACS Octo Dissociator. Tumor dissociation kit (catalog #130-096-730; Miltenyi Biotec) was used per the manufacturer's protocol for tumor dissociation, and a mouse spleen dissociation kit (catalog 130-095-926; Miltenyi Biotec) was used for dissociation of spleen per the manufacturer's protocol into single-cell suspension. Several rounds of red blood cell (RBC) lysis was performed on blood and single-cell suspensions from tumors and spleens, using RBC Lysing Buffer Hybri-Max (catalog R7757-100ML; Sigma-Aldrich). Next, cells were washed twice with PBS and stained with Zombie UV Fixable Viability Dye (catalog #423,108; BioLegend) diluted 1:500 in PBS. Cells were then washed once with PBS, followed by another wash with fluorescence-activated cell sorting (FACS) buffer (PBS + 2% FBS). Cells were then blocked with anti-mouse CD16/32 antibody (catalog #101,320, clone #93; BioLegend) in FACS buffer for 10 min and then stained with a cocktail of PerCP/Cyanine5.5 anti-mouse CD45 antibody (catalog #103,132, clone #30-F11; BioLegend), PE anti-mouse CD8a antibody (catalog #100,708, clone #53–6.7; BioLegend), APC anti-mouse CD279 (PD-1) antibody (catalog #135,210, clone #29F.1A12; BioLegend), PE/Cyanine7 anti-mouse CD274 (B7-H1, PD-L1) antibody (catalog #124,314, clone #10F.9G2; BioLegend), and APC/Cyanine7 anti-mouse CD4 antibody (catalog #100,526, clone #RM4-5; BioLegend) antibody for 20 min at room temperature in the dark. After staining, cells were fixed with 4% paraformaldehyde and stored at 4°C overnight. The following day, samples were run on a BD LSRFortessa. FlowJo software was used to analyze the flow cytometry data.

qPCR for quantification of virus and anti-PD-L1

Frozen tumor samples were divided into two halves; one-half was weighed and used for RNA isolation using the RNeasy Mini Kit (catalog #74,104; Qiagen), and another half was homogenized in RIPA buffer for western blot analysis. Next, cDNA was synthesized using 1,000 ng RNA using the QuantiTect Reverse Transcription Kit (catalog #205311; Qiagen) following the manufacturer's instructions. cDNA was synthesized in a total volume of 20 μL, of which 4 μL was used to run qPCR using TaqMan-based assay. Primers-probe specific for the viral gene *F4L* was used for quantification of virus genome, and primers-probe specific for

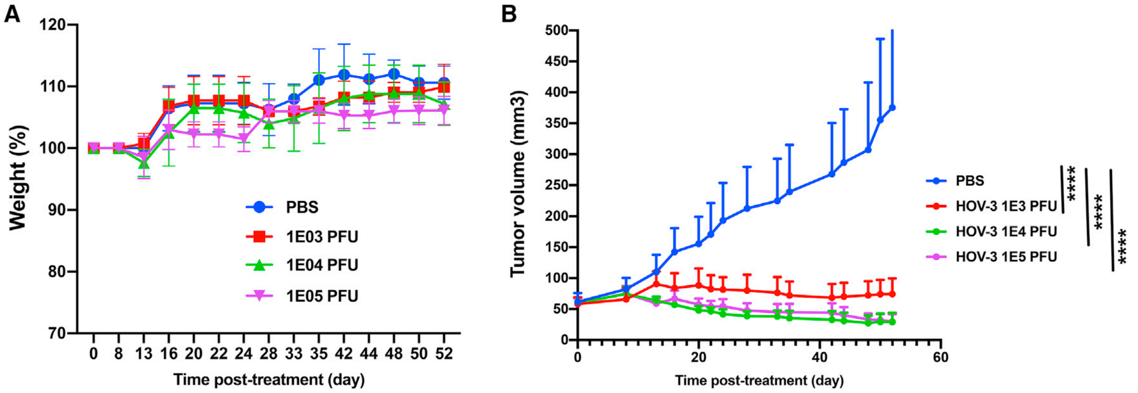

Figure 11. Safety and anti-tumor efficacy in a xenograft model

(A) Mice (n = 5/group) were weighed two or three times a week, and the average body weight for each group ± SEM is plotted. (B) Tumors were measured two to three times a week using digital calipers, and tumor volume was calculated using the equation: volume = length × $(\text{breadth})^2/2$. Average volume + SEM is plotted. ****$p < 0.0001$, two-way ANOVA with Dunnett's multiple-comparisons test.

the anti-PD-L1 gene spanning the FLAG tag sequence was used for detection of virus-encoded anti-PD-L1. Primers-probe set specific for mouse GAPDH was used as an internal control for further normalization of the data. All primers and probes, except for GAPDH, were self-designed and purchased from Integrated DNA Technologies. Primers-probe set for GAPDH was purchased from Qiagen. Ct values for GAPDH were used for normalization of anti-PD-L1 expression levels.

To quantify virus genome copy number, a standard curve was generated using a known amount plasmid DNA-containing viral sequence (*F4L* gene). Number of virus genome was calculated on the basis of the standard curve generated using the built-in function of qPCR machine.

To determine the stability of CF33-hNIS-anti-PD-L1 at room temperature compared with ice, two vials of CF33-hNIS-anti-PD-L1 virus (one vial at a concentration of 1×10^8 PFU/mL and one vial at a concentration of 1×10^6 PFU/mL) were removed from the −80°C freezer and thawed at room temperature. After thawing, each vial of virus was sonicated for 1 min in ice water, and 50 μL aliquots were taken from each vial for immediate titration, which would be used as the control. The remaining virus at each concentration were divided into two microcentrifuge tubes (450 μL/tube), and one tube was left at room temperature and another tube was placed on ice. From each tube, aliquots of 50 μL virus were taken at 30 min intervals for up to 4 h, and the aliquots of virus were used immediately to infect CV-1 cells for titration at each time point. Forty-eight hours after infection of CV-1 cells, plaques were counted, and titers of virus at each time point were determined using viral plaque assay on CV-1 cells and plotted.

Stability of CF33-hNIS-anti-PD-L1 after freeze-thaw cycles

Two vials of CF33-hNIS-anti-PD-L1 virus at a concentration of 1E08 or 1E06 PFU/mL were removed from the −80°C freezer and thawed for either 30 min or 1 h at room temperature. After thawing, an aliquot of 50 μL was taken from each vial and used immediately for infecting CV-1 cells, and the original vials were immediately returned

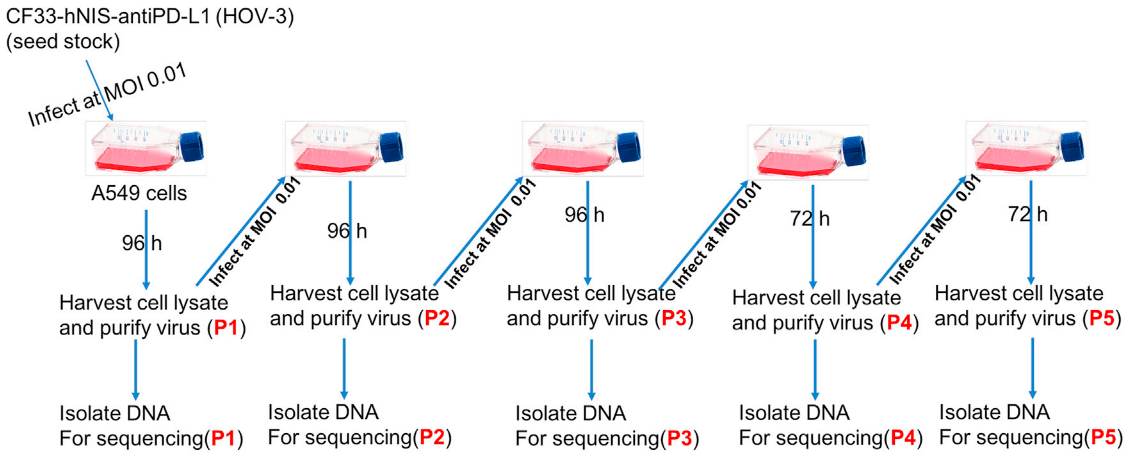

Figure 12. Schema showing serial passaging of CF33-hNIS-anti-PD-L1 and DNA isolation for sequencing

to −80°C freezer. The vials were kept in the −80°C freezer for at least 1 h and again thawed for 30 min or 1 h at room temperature, and aliquots were taken and immediately used to infect CV-1 cells. These freeze-thaw cycles were continued for up to 5 cycles, and virus titers were determined after each thaw by plaque assay on CV-1 cells.

Compatibility of CF33-hNIS-anti-PD-L1 with syringe and needle

Vials containing 1 mL CF33-hNIS-anti-PD-L1 virus (lab stocks) at a concentration of 1E08 or 1E06 PFU/mL were taken out of the −80°C freezer and thawed at room temperature. The stocks were then diluted 1:100 with PBS to obtain 1E06 or 1E04 PFU/mL. Next, 10 mL of diluted viruses were loaded in 10 mL sterile syringes (catalog #309604, lot #6291821; BD Biosciences) fitted with sterile 25G needles (catalog #305125, lot #0274523; BD Biosciences). The plunger of the syringe was slowly pushed to eject virus solution from the syringe, and the ejected viruses were collected in a 15 mL conical tube and were immediately used for titration by plaque assay.

SUPPLEMENTAL INFORMATION

Supplemental information can be found online at https://doi.org/10.1016/j.omtm.2021.12.002.

ACKNOWLEDGMENTS

S.C. is supported by research grant from the California Breast Cancer Research Program (CBCRP).

AUTHOR CONTRIBUTIONS

S.C., A.Y., Z.Z., J.L., H.V., S.K., and T.O. performed the experiments. Y.Z. and X.W. performed sequencing and bio-informatics analyses. S.C., Y.W., S.G.W., S.F., L.C., M.C., N.E., and Y.F. were involved in study design. S.C. wrote the manuscript.

DECLARATION OF INTERESTS

Y.F. is an adviser to Imugene Ltd. and receives royalties from Imugene and Merck. S.C. is a consultant to Imugene Ltd. All other authors declare no competing interests.

REFERENCES

1. Chaurasiya, S., Fong, Y., and Warner, S.G. (2021). Oncolytic virotherapy for cancer: clinical experience. Biomedicines *9*. https://doi.org/10.3390/biomedicines9040419.
2. Conry, R.M., Westbrook, B., McKee, S., and Norwood, T.G. (2018). Talimogene laherparepvec: first in class oncolytic virotherapy. Hum. Vaccin. Immunother. *14*, 839–846. https://doi.org/10.1080/21645515.2017.1412896.
3. Kaufman, H.L., Kohlhapp, F.J., and Zloza, A. (2016). Oncolytic viruses: a new class of immunotherapy drugs. Nat. Rev. Drug Discov. *15*, 660. https://doi.org/10.1038/nrd.2016.178.
4. Chaurasiya, S., Chen, N.G., and Fong, Y. (2018). Oncolytic viruses and immunity. Curr. Opin. Immunol. *51*, 83–90. https://doi.org/10.1016/j.coi.2018.03.008.
5. Puzanov, I., Milhem, M.M., Minor, D., Hamid, O., Li, A., Chen, L., Chastain, M., Gorski, K.S., Anderson, A., Chou, J., et al. (2016). Talimogene laherparepvec in combination with ipilimumab in previously untreated, unresectable stage IIIB-IV melanoma. J. Clin. Oncol. *34*, 2619–2626. https://doi.org/10.1200/JCO.2016.67.1529.
6. Ribas, A., Dummer, R., Puzanov, I., VanderWalde, A., Andtbacka, R.H.I., Michielin, O., Olszanski, A.J., Malvehy, J., Cebon, J., Fernandez, E., et al. (2017). Oncolytic virotherapy promotes intratumoral T cell infiltration and improves anti-PD-1 immunotherapy. Cell *170*, 1109–1119 e1110. https://doi.org/10.1016/j.cell.2017.08.027.
7. Andrews, A. (2015). Treating with checkpoint inhibitors-figure $1 million per patient. Am. Health Drug Benefits *8*, 9.
8. Orloff, M. (2016). Spotlight on talimogene laherparepvec for the treatment of melanoma lesions in the skin and lymph nodes. Oncolytic Virother. *5*, 91–98. https://doi.org/10.2147/OV.S99532.
9. Woo, Y., Zhang, Z., Yang, A., Chaurasiya, S., Park, A.K., Lu, J., Kim, S.I., Warner, S.G., Von Hoff, D., and Fong, Y. (2020). Novel chimeric immuno-oncolytic virus CF33-hNIS-antiPDL1 for the treatment of pancreatic cancer. J. Am. Coll. Surg. https://doi.org/10.1016/j.jamcollsurg.2019.12.027.
10. Chaurasiya, S., Chen, N.G., Lu, J., Martin, N., Shen, Y., Kim, S.I., Warner, S.G., Woo, Y., and Fong, Y. (2019). A chimeric poxvirus with J2R (thymidine kinase) deletion shows safety and anti-tumor activity in lung cancer models. Cancer Gene Ther. https://doi.org/10.1038/s41417-019-0114-x.
11. Lassmann, T. (2019). Kalign 3: multiple sequence alignment of large data sets. Bioinformatics. https://doi.org/10.1093/bioinformatics/btz795.
12. Johnston, J.B., and McFadden, G. (2004). Technical knockout: understanding poxvirus pathogenesis by selectively deleting viral immunomodulatory genes. Cell. Microbiol. *6*, 695–705. https://doi.org/10.1111/j.1462-5822.2004.00423.x.
13. Dohm, J.C., Peters, P., Stralis-Pavese, N., and Himmelbauer, H. (2020). Benchmarking of long-read correction methods. NAR Genom. Bioinform. *2*, lqaa037. https://doi.org/10.1093/nargab/lqaa037.
14. Israely, T., Paran, N., Erez, N., Cherry, L., Tamir, H., Achdout, H., Politi, B., Israeli, O., Zaide, G., Cohen-Gihon, I., et al. (2019). Differential response following infection of mouse CNS with virulent and attenuated vaccinia virus strains. Vaccines (Basel) *7*. https://doi.org/10.3390/vaccines7010019.
15. Li, Z., Rubin, S.A., Taffs, R.E., Merchlinsky, M., Ye, Z., and Carbone, K.M. (2004). Mouse neurotoxicity test for vaccinia-based smallpox vaccines. Vaccine *22*, 1486–1493. https://doi.org/10.1016/j.vaccine.2003.10.022.
16. Chaurasiya, S., Yang, A., Kang, S., Lu, J., Kim, S.I., Park, A.K., Sivanandam, V., Zhang, Z., Woo, Y., Warner, S.G., and Fong, Y. (2020). Oncolytic poxvirus CF33-hNIS-DeltaF14.5 favorably modulates tumor immune microenvironment and works synergistically with anti-PD-L1 antibody in a triple-negative breast cancer model. Oncoimmunology *9*, 1729300. https://doi.org/10.1080/2162402X.2020.1729300.
17. Flores, E.B., Aksoy, B.A., and Bartee, E. (2020). Initial dose of oncolytic myxoma virus programs durable antitumor immunity independent of in vivo viral replication. J. Immunother. Cancer *8*. https://doi.org/10.1136/jitc-2020-000804.
18. Pugalenthi, A., Mojica, K., Ady, J.W., Johnsen, C., Love, D., Chen, N.G., Aguilar, R.J., Szalay, A.A., and Fong, Y. (2015). Recombinant vaccinia virus GLV-1h68 is a promising oncolytic vector in the treatment of cholangiocarcinoma. Cancer Gene Ther. *22*, 591–596. https://doi.org/10.1038/cgt.2015.60.
19. Brun, J., Mahoney, D.J., Le Boeuf, F., Lefebvre, C., Sanaei, C.A., Falls, T., McCart, J.A., and Stojdl, D.F. (2013). Oncolytic Vaccinia virus safely and effectively treats skin tumors in mouse models of xeroderma pigmentosum. Int. J. Cancer *132*, 726–731. https://doi.org/10.1002/ijc.27695.
20. Guse, K., Sloniecka, M., Diaconu, I., Ottolino-Perry, K., Tang, N., Ng, C., Le Boeuf, F., Bell, J.C., McCart, J.A., Ristimaki, A., et al. (2010). Antiangiogenic arming of an oncolytic vaccinia virus enhances antitumor efficacy in renal cell cancer models. J. Virol. *84*, 856–866. https://doi.org/10.1128/JVI.00692-09.
21. O'Leary, M.P., Choi, A.H., Kim, S.I., Chaurasiya, S., Lu, J., Park, A.K., Woo, Y., Warner, S.G., Fong, Y., and Chen, N.G (2018). Novel oncolytic chimeric orthopoxvirus causes regression of pancreatic cancer xenografts and exhibits abscopal effect at a single low dose. J. Transl. Med. *16*, 110. https://doi.org/10.1186/s12967-018-1483-x.
22. Cotter, C.A., Earl, P.L., Wyatt, L.S., and Moss, B. (2015). Preparation of cell cultures and vaccinia virus stocks. Curr. Protoc. Microbiol. *39*, 14A 13 11–14A 13 18. https://doi.org/10.1002/9780471729259.mc14a03s39.
23. Moss, B. (2013). Poxvirus DNA replication. Cold Spring Harb. Perspect. Biol. *5*. https://doi.org/10.1101/cshperspect.a010199.
24. Johnston, J.B., and McFadden, G. (2003). Poxvirus immunomodulatory strategies: current perspectives. J. Virol. *77*, 6093–6100. https://doi.org/10.1128/jvi.77.11.6093-6100.2003.

25. Shchelkunov, S.N. (2012). Orthopoxvirus genes that mediate disease virulence and host tropism. Adv. Virol. *2012*, 524743. https://doi.org/10.1155/2012/524743.

26. Seet, B.T., Johnston, J.B., Brunetti, C.R., Barrett, J.W., Everett, H., Cameron, C., Sypula, J., Nazarian, S.H., Lucas, A., and McFadden, G. (2003). Poxviruses and immune evasion. Annu. Rev. Immunol. *21*, 377–423. https://doi.org/10.1146/annurev.immunol.21.120601.141049.

27. McFadden, G. (2005). Poxvirus tropism. Nat. Rev. Microbiol. *3*, 201–213. https://doi.org/10.1038/nrmicro1099.

28. Werden, S.J., Rahman, M.M., and McFadden, G. (2008). Poxvirus host range genes. Adv. Virus Res. *71*, 135–171. https://doi.org/10.1016/S0065-3527(08)00003-1.

29. Deng, R., Huang, M., Wang, J., Huang, Y., Yang, J., Feng, J., and Wang, X. (2006). PTreeRec: phylogenetic Tree Reconstruction based on genome BLAST distance. Comput. Biol. Chem. *30*, 300–302. https://doi.org/10.1016/j.compbiolchem.2006.04.003.

30. Tamura, K., Nei, M., and Kumar, S. (2004). Prospects for inferring very large phylogenies by using the neighbor-joining method. Proc. Natl. Acad. Sci. U S A *101*, 11030–11035. https://doi.org/10.1073/pnas.0404206101.

OMTM, Volume *24*

Supplemental information

A comprehensive preclinical study supporting clinical trial of oncolytic chimeric poxvirus CF33-hNIS-anti-PD-L1 to treat breast cancer

Shyambabu Chaurasiya, Annie Yang, Zhifang Zhang, Jianming Lu, Hannah Valencia, Sang-In Kim, Yanghee Woo, Suanne G. Warner, Tove Olafsen, Yuqi Zhao, Xiwei Wu, Seymour Fein, Linda Cheng, Maria Cheng, Nicholas Ede, and Yuman Fong

Table 1: List of CF33 genes involved in entry, attachment, replication and pathogenesis, and their origins.

Categories	Descriptions	Genes	Origins
Prevention of superinfection	prevents superinfection	A33R	AS
	prevents superinfection	A36R	WR or Rabbitpox
Precursor Metabolism	dNTP synthesis	F4L	IHD
	dNTP synthesis	A48R	IHD
	dNTP synthesis	I4L	WR
	dNTP synthesis	J2R	WR or IHD or Rabbitpox
Pathogenesis	Semaphorin-like	A39R	AS
	inhibitor of antigen presentation by MHC II	A35R	AS or Lister or Rabbitpox
	complement control	C21L	CL or WR or IHD or Lister or Rabbitpox
	complement control	C21L	CL or WR or IHD or Lister or Rabbitpox
	TLR inhibitor	A46R	IHD
	TLR inhibitor	A52R	IHD
	anti-apoptotic protein serpin	SPI-2/CrmA	IHD
	Type 1 IFN-R mimic	B18R	IHD
	inhibits IL-18	C12L	IHD
	eIF2a-mimic; IFN I resistance	K3L	Lederle
	Inhibition of NF-B and IRF3	K7R	Lederle
	Inhibition of apoptosis	N1L	Lederle or WR or IHD or Lister or Rabbitpox
	EGF-like growth factor	C11R	WR
	Type 2 IFN-R mimic	B8R	WR or IHD
	dsRNA binding protein; block IFN I	E3L	WR or IHD or Rabbitpox
Entry	Entry	A21L	AS or IHD or Lister or Rabbitpox
	Entry	G3L	Lederle or AS or WR or IHD or Lister or Rabbitpox
	Entry	H2R	Lederle or AS or WR or Lister
	Entry	A28L	Lederle or WR
	Entry	F9L	Lederle or WR or IHD or Lister
	Entry	L5R	Lederle or WR or IHD or Lister
	Entry	I2L	Lister
	Entry	J5L	Lister
	Entry	A16L	Rabbitpox
	Entry	G9R	WR
	Entry	L1R	WR
DNA replication	FEN1 like nuclease	G5R	AS
	DNA polymerase	E9L	AS or IHD or Lister or Rabbitpox
	Telomere binding protein 1	I1L	AS or Lister
	Processivity factor	A20R	AS or Lister
	ATPase	A32L	IHD
	DNA ligase	A50R	IHD
	Protein Kinase	B1R	IHD
	Uracil. DNA glkycosylase	D4R	Lederle or WR

Table 1 (Continued):

DNA replication	Topoisomerase	H6R	Rabbitpox
	Single stranded DNA binding protein	I3L	WR
	Helicase-Primase	D5R	WR
	Holiday junction resolvase	A22R	WR
Cell-cell fusion	cell-cell fusion	A56R	IHD
	cell-cell fusion	K2L/SPI-3	WR
Attachment	Attachment	A26L	Lister
	Attachment	H3L	WR
	Attachment	D8L	WR
	Attachment	A27L	WR

Table 2: Summary of Study Design – Toxicology Groups

Group	Number of Mice	Animal Number (M/F)	Tumor Induc-tion	Test Article Treatment		In-life Procedure	Termination	Terminal Procedure
1	6M /6F	101-106 /151-156	Yes	IT 1X Day 0	Control Article (Vehicle)	Clinical Observations: 1x daily; Body Condition Scoring: weekly; Body Weights: weekly; Food Consumption: weekly; Tumor measurement: 2x weekly	24 Hr	Clinical pathology; Necropsy; organ weights; histopathology
	6M /6F	107-112 /157-162					Day 7	
	8M /8F	113-120 /163-170					Day 21	
2	6M /6F	201-206 /251-256			CF33-hNIS-antiPDL1 1E03 PFU		24 Hr	
	6M /6F	207-212 /257-262					Day 7	
	8M /8F	213-220 /263-270					Day 21	
3	6M /6F	301-306 /351-356			CF33-hNIS-antiPDL1 1E04 PFU		24 Hr	
	6M /6F	307-312 /357-362					Day 7	
	8M /8F	313-320 /363-370					Day 21	
4	6M /6F	401-406 /451-456			CF33-hNIS-antiPDL1 1E05 PFU		24 Hr	
	6M /6F	407-412 /457-462					Day 7	
	8M /8F	413-420 /463-470					Day 21	
5	6M /6F	501-506 /551-556		IT 6X Day 0-12	Control Article (Vehicle)		Day 12	
	8M /8F	507-514 /557-564					Day 21	
6	6M /6F	601-606 /651-656			CF33-hNIS-antiPDL1 1E03 PFU		Day 12	
	8M /8F	607-614 /657-664					Day 21	
7	6M /6F	701-706 /751-756			CF33-hNIS-antiPDL1 1E05 PFU		Day 12	
	8M /8F	707-714 /757-764					Day 21	

Table 3: Summary of Study Design – Biodistribution Groups

Group	Number of Mice	Animal Number (F)	Tumor Induction	Test Article Treatment		In-life Procedure	Termination	Terminal Procedure
**8**	5F	851-855	Yes	IT 1X Day0	Control Article (Vehicle)	Clinical Observations: 1x daily; Body Condition Scoring: weekly; Body Weights: weekly; Food Consumption: weekly; Tumor measurement: 2x weekly	Day 7	Terminal blood and tissue collection for viral plaques and PCR
	8F	856-863					Day 21	
**9**	5F	951-955			CF33-hNIS-antiPDL1 1E03 PFU		Day 7	
	8F	956-963					Day 21	
**10**	5F	1051-1055			CF33-hNIS-antiPDL1 1E04 PFU		Day 7	
	8F	1056-1063					Day 21	
**11**	5F	1151-1155			CF33-hNIS-antiPDL1 1E05 PFU		Day 7	
	8F	1156-1163					Day 21	
**12**	5F	1251-1255		IT 6X Day 0-12	Control Article (Vehicle)		Day 12	
	8F	1256-1263			0.1 mL		Day 21	
**13**	5F	1351-1355			CF33-hNIS-antiPDL1 1E03 PFU		Day 12	
	8F	1356-1363					Day 21	
**14**	5F	1451-1455			CF33-hNIS-antiPDL1 1E05 PFU		Day 12	
	8F	1456-1463					Day 21	

Table 4: Summary of Study Design – Troponin Groups

Group	Number of Mice	Animal Number (M/F)	Tumor Induction	Test Article Treatment		In-life Procedure	Termination	Terminal Procedure
**15**	5F	1551-1555	Yes	IT 1X Day0	Control Article (Vehicle)	Clinical Observations: 1x daily; Body Condition Scoring: weekly; Body Weights: weekly; Food Consumption: weekly; Tumor measurement: 2x weekly	24 Hr	Terminal blood collection for troponins
	8F	1556-1563					Day 14	
**16**	5F	1651-1655			CF33-hNIS-antiPDL1 1E05 PFU		24 Hr	
	8F	1656-1663					Day 14	
**17**	8F	1751-1758		IT 6X Day 0-12	Control Article (Vehicle)		Day 21	
**18**	8F	1851-1858			CF33-hNIS-antiPDL1 1E03 PFU			
**19**	8F	1851-1858			CF33-hNIS-antiPDL1 1E05 PFU			

Table 5: Summary of Study Design – Cytokine Groups

Group	Number of Mice	Animal Number (M/F)	Tumor Inducti on	Test Article Treatment		In-life Procedure	Termination	Terminal Procedure
	5F	2051-2055			Control Article (Vehicle)		3 Hr	
**20**	5F	2056-2060					Day 7	
	8F	2061-2068					Day 14	
	5F	2151-2155			CF33-hNIS-antiPDL1 1E03 PFU		3 Hr	
**21**	5F	2156-2160		IT 1X Day0		Clinical Observations: 1x daily; Body Condition Scoring: weekly; Body Weights: weekly; Food Consumption: weekly; Tumor measurement: 2x weekly	Day 7	
	8F	2161-2168					Day 14	
	5F	2251-2255			CF33-hNIS-antiPDL1 1E03 PFU		3 Hr	
**22**	5F	2256-2260					Day 7	Terminal blood collection for cytokines
	8F	2261-2268	Yes				Day 14	
**23**	8F	2351-2358			Control Article (Vehicle)			
**24**	8F	2451-2458		IT 6X Day 0-12	CF33-hNIS-antiPDL1 1E03 PFU		Day 21	
**25**	8F	2551-2558			CF33-hNIS-antiPDL1 1E05 PFU			

Table 6: Sample Concentrations of IFN-γ in Mouse Serum

Run ID	Subject	Day (Nominal)	Concentration (pg/mL)	Concentration Units	Dilution Factor	Biological Matrix
36	2051	3 Hr	0.448	pg/mL	1	Serum
36	2052	3 Hr	BLQ<(0.187)	pg/mL	1	Serum
36	2053	3 Hr	BLQ<(0.187)	pg/mL	1	Serum
36	2054	3 Hr	BLQ<(0.187)	pg/mL	1	Serum
36	2055	3 Hr	BLQ<(0.187)	pg/mL	1	Serum
36	2056	7	BLQ<(0.187)	pg/mL	1	Serum
36	2057	7	0.313	pg/mL	1	Serum
36	2058	7	BLQ<(0.187)	pg/mL	1	Serum
36	2059	7	0.316	pg/mL	1	Serum
36	2060	7	0.228	pg/mL	1	Serum
36	2061	14	0.31	pg/mL	1	Serum
36	2062	14	0.32	pg/mL	1	Serum
36	2063	14	0.498	pg/mL	1	Serum
36	2064	14	0.639	pg/mL	1	Serum
36	2065	14	0.625	pg/mL	1	Serum
36	2066	14	0.434	pg/mL	1	Serum
36	2067	14	0.222	pg/mL	1	Serum
36	2068	14	0.384	pg/mL	1	Serum
36	2151	3 Hr	0.331	pg/mL	1	Serum
36	2152	3 Hr	0.207	pg/mL	1	Serum
36	2153	3 Hr	0.316	pg/mL	1	Serum
36	2154	3 Hr	BLQ<(0.187)	pg/mL	1	Serum
36	2155	3 Hr	0.187	pg/mL	1	Serum
36	2156	7	0.39	pg/mL	1	Serum
36	2157	7	BLQ<(0.187)	pg/mL	1	Serum
36	2158	7	0.365	pg/mL	1	Serum
36	2159	7	0.23	pg/mL	1	Serum
36	2160	7	0.217	pg/mL	1	Serum
36	2161	14	0.28	pg/mL	1	Serum
36	2162	14	0.26	pg/mL	1	Serum
36	2163	14	0.308	pg/mL	1	Serum
36	2164	14	BLQ<(0.187)	pg/mL	1	Serum
36	2165	14	0.481	pg/mL	1	Serum
36	2166	14	0.328	pg/mL	1	Serum
36	2167	14	0.35	pg/mL	1	Serum
41	2168	14	0.432	pg/mL	1	Serum
41	2251	3 Hr	0.203	pg/mL	1	Serum
41	2252	3 Hr	0.395	pg/mL	1	Serum
41	2253	3 Hr	BLQ<(0.187)	pg/mL	1	Serum
41	2254	3 Hr	0.279	pg/mL	1	Serum
41	2255	3 Hr	0.212	pg/mL	1	Serum

41	2256	7	0.231	pg/mL	1	Serum
41	2257	7	0.944	pg/mL	1	Serum

Table 6 (Continued)

41	2258	7	0.344	pg/mL	1	Serum
41	2259	7	0.381	pg/mL	1	Serum
41	2260	7	0.202	pg/mL	1	Serum
41	2261	14	0.401	pg/mL	1	Serum
41	2262	14	0.353	pg/mL	1	Serum
41	2263	14	0.298	pg/mL	1	Serum
41	2264	14	0.58	pg/mL	1	Serum
41	2265	14	0.207	pg/mL	1	Serum
41	2266	14	BLQ<(0.187)	pg/mL	1	Serum
41	2267	14	0.244	pg/mL	1	Serum
41	2268	14	0.401	pg/mL	1	Serum
41	2351	19	BLQ<(0.187)	pg/mL	1	Serum
41	2352	19	BLQ<(0.187)	pg/mL	1	Serum
41	2353	19	BLQ<(0.187)	pg/mL	1	Serum
41	2354	19	0.195	pg/mL	1	Serum
41	2355	14	0.874	pg/mL	1	Serum
41	2356	19	0.201	pg/mL	1	Serum
41	2357	21	0.215	pg/mL	1	Serum
41	2358	19	0.291	pg/mL	1	Serum
41	2451	19	BLQ<(0.187)	pg/mL	1	Serum
41	2452	14	0.314	pg/mL	1	Serum
41	2453	21	0.239	pg/mL	1	Serum
41	2454	14	0.273	pg/mL	1	Serum
41	2455	14	0.207	pg/mL	1	Serum
41	2456	14	0.326	pg/mL	1	Serum
41	2457	19	0.319	pg/mL	1	Serum
41	2458	14	0.309	pg/mL	1	Serum
31	2551	19	BLQ<(0.187)	pg/mL	1	Serum
31	2552	19	BLQ<(0.187)	pg/mL	1	Serum
31	2553	14	0.261	pg/mL	1	Serum
31	2554	19	0.196	pg/mL	1	Serum
31	2555	19	BLQ<(0.187)	pg/mL	1	Serum
31	2556	21	0.282	pg/mL	1	Serum
31	2557	19	BLQ<(0.187)	pg/mL	1	Serum
31	2558	21	0.296	pg/mL	1	Serum

Table 7: Sample Concentrations of IL-1β in Mouse Serum

Run ID	Subject	Day Nominal	Concentration (pg/mL)	Concentration Units	Dilution Factor	Biological Matrix
37	2051	3 Hr	BLQ<(0.374)	pg/mL	1	Serum
37	2052	3 Hr	BLQ<(0.374)	pg/mL	1	Serum
37	2053	3 Hr	BLQ<(0.374)	pg/mL	1	Serum
37	2054	3 Hr	BLQ<(0.374)	pg/mL	1	Serum
37	2055	3 Hr	BLQ<(0.374)	pg/mL	1	Serum
37	2056	7	BLQ<(0.374)	pg/mL	1	Serum
37	2057	7	BLQ<(0.374)	pg/mL	1	Serum
37	2058	7	BLQ<(0.374)	pg/mL	1	Serum
37	2059	7	BLQ<(0.374)	pg/mL	1	Serum
37	2060	7	BLQ<(0.374)	pg/mL	1	Serum
37	2061	14	BLQ<(0.374)	pg/mL	1	Serum
37	2062	14	BLQ<(0.374)	pg/mL	1	Serum
37	2063	14	BLQ<(0.374)	pg/mL	1	Serum
37	2064	14	BLQ<(0.374)	pg/mL	1	Serum
37	2065	14	BLQ<(0.374)	pg/mL	1	Serum
37	2066	14	BLQ<(0.374)	pg/mL	1	Serum
37	2067	14	BLQ<(0.374)	pg/mL	1	Serum
37	2068	14	BLQ<(0.374)	pg/mL	1	Serum
37	2151	3 Hr	BLQ<(0.374)	pg/mL	1	Serum
37	2152	3 Hr	BLQ<(0.374)	pg/mL	1	Serum
37	2153	3 Hr	BLQ<(0.374)	pg/mL	1	Serum
37	2154	3 Hr	BLQ<(0.374)	pg/mL	1	Serum
37	2155	3 Hr	BLQ<(0.374)	pg/mL	1	Serum
37	2156	7	BLQ<(0.374)	pg/mL	1	Serum
37	2157	7	BLQ<(0.374)	pg/mL	1	Serum
37	2158	7	BLQ<(0.374)	pg/mL	1	Serum
37	2159	7	BLQ<(0.374)	pg/mL	1	Serum
37	2160	7	BLQ<(0.374)	pg/mL	1	Serum
37	2161	14	BLQ<(0.374)	pg/mL	1	Serum
37	2162	14	BLQ<(0.374)	pg/mL	1	Serum
37	2163	14	BLQ<(0.374)	pg/mL	1	Serum
37	2164	14	BLQ<(0.374)	pg/mL	1	Serum
37	2165	14	BLQ<(0.374)	pg/mL	1	Serum
37	2166	14	BLQ<(0.374)	pg/mL	1	Serum
37	2167	14	BLQ<(0.374)	pg/mL	1	Serum
42	2168	14	BLQ<(0.374)	pg/mL	1	Serum
42	2251	3 Hr	BLQ<(0.374)	pg/mL	1	Serum
42	2252	3 Hr	BLQ<(0.374)	pg/mL	1	Serum
42	2253	3 Hr	BLQ<(0.374)	pg/mL	1	Serum
42	2254	3 Hr	BLQ<(0.374)	pg/mL	1	Serum
42	2255	3 Hr	BLQ<(0.374)	pg/mL	1	Serum

42	2256	7	BLQ<(0.374)	pg/mL	1	Serum

Table 7. (continued)

42	2257	7	BLQ<(0.374)	pg/mL	1	Serum
42	2258	7	BLQ<(0.374)	pg/mL	1	Serum
42	2259	7	BLQ<(0.374)	pg/mL	1	Serum
42	2260	7	BLQ<(0.374)	pg/mL	1	Serum
42	2261	14	BLQ<(0.374)	pg/mL	1	Serum
42	2262	14	BLQ<(0.374)	pg/mL	1	Serum
42	2263	14	BLQ<(0.374)	pg/mL	1	Serum
42	2264	14	BLQ<(0.374)	pg/mL	1	Serum
42	2265	14	BLQ<(0.374)	pg/mL	1	Serum
42	2266	14	BLQ<(0.374)	pg/mL	1	Serum
42	2267	14	BLQ<(0.374)	pg/mL	1	Serum
42	2268	14	BLQ<(0.374)	pg/mL	1	Serum
42	2351	19	BLQ<(0.374)	pg/mL	1	Serum
42	2352	19	BLQ<(0.374)	pg/mL	1	Serum
42	2353	19	BLQ<(0.374)	pg/mL	1	Serum
42	2354	19	BLQ<(0.374)	pg/mL	1	Serum
42	2355	14	BLQ<(0.374)	pg/mL	1	Serum
42	2356	19	BLQ<(0.374)	pg/mL	1	Serum
42	2357	21	BLQ<(0.374)	pg/mL	1	Serum
42	2358	19	BLQ<(0.374)	pg/mL	1	Serum
42	2451	19	BLQ<(0.374)	pg/mL	1	Serum
42	2452	14	BLQ<(0.374)	pg/mL	1	Serum
42	2453	21	BLQ<(0.374)	pg/mL	1	Serum
42	2454	14	BLQ<(0.374)	pg/mL	1	Serum
42	2455	14	BLQ<(0.374)	pg/mL	1	Serum
42	2456	14	BLQ<(0.374)	pg/mL	1	Serum
42	2457	19	BLQ<(0.374)	pg/mL	1	Serum
42	2458	14	BLQ<(0.374)	pg/mL	1	Serum
32	2551	19	BLQ<(0.374)	pg/mL	1	Serum
32	2552	19	BLQ<(0.374)	pg/mL	1	Serum
32	2553	14	BLQ<(0.374)	pg/mL	1	Serum
32	2554	19	BLQ<(0.374)	pg/mL	1	Serum
32	2555	19	BLQ<(0.374)	pg/mL	1	Serum
32	2556	21	BLQ<(0.374)	pg/mL	1	Serum
32	2557	19	BLQ<(0.374)	pg/mL	1	Serum
32	2558	21	BLQ<(0.374)	pg/mL	1	Serum

Table 8: Sample Concentrations of IL-6 in Mouse Serum

Run ID	Subject	Day (Nominal)	Concentration (pg/mL)	Concentration Units	Dilution Factor	Biological Matrix
38	2051	3 Hr	8.06	pg/mL	1	Serum
38	2052	3 Hr	3.23	pg/mL	1	Serum
38	2053	3 Hr	1.96	pg/mL	1	Serum
38	2054	3 Hr	2.86	pg/mL	1	Serum
38	2055	3 Hr	11.9	pg/mL	1	Serum
38	2056	7	6.21	pg/mL	1	Serum
38	2057	7	13.8	pg/mL	1	Serum
38	2058	7	7.01	pg/mL	1	Serum
38	2059	7	9.59	pg/mL	1	Serum
38	2060	7	28.4	pg/mL	1	Serum
38	2061	14	20.6	pg/mL	1	Serum
38	2062	14	37.6	pg/mL	1	Serum
38	2063	14	27.7	pg/mL	1	Serum
38	2064	14	25.6	pg/mL	1	Serum
38	2065	14	20.1	pg/mL	1	Serum
38	2066	14	89.1	pg/mL	1	Serum
38	2067	14	2.52	pg/mL	1	Serum
38	2068	14	33.8	pg/mL	1	Serum
38	2151	3 Hr	4.74	pg/mL	1	Serum
38	2152	3 Hr	4.21	pg/mL	1	Serum
38	2153	3 Hr	2.54	pg/mL	1	Serum
38	2154	3 Hr	6.09	pg/mL	1	Serum
38	2155	3 Hr	3.14	pg/mL	1	Serum
38	2156	7	13.2	pg/mL	1	Serum
38	2157	7	14.3	pg/mL	1	Serum
38	2158	7	9.41	pg/mL	1	Serum
38	2159	7	27.4	pg/mL	1	Serum
38	2160	7	7.58	pg/mL	1	Serum
38	2161	14	27.2	pg/mL	1	Serum
38	2162	14	50.7	pg/mL	1	Serum
38	2163	14	90.1	pg/mL	1	Serum
38	2164	14	23.2	pg/mL	1	Serum
38	2165	14	28.5	pg/mL	1	Serum
38	2166	14	75.3	pg/mL	1	Serum
38	2167	14	34.8	pg/mL	1	Serum
43	2168	14	100	pg/mL	1	Serum
43	2251	3 Hr	3.57	pg/mL	1	Serum
43	2252	3 Hr	12.6	pg/mL	1	Serum
43	2253	3 Hr	4.22	pg/mL	1	Serum
43	2254	3 Hr	4.48	pg/mL	1	Serum

43	2255	3 Hr	4.3	pg/mL	1	Serum

Table 8. (continued)

43	2256	7	9.93	pg/mL	1	Serum
43	2257	7	17.1	pg/mL	1	Serum
43	2258	7	57.8	pg/mL	1	Serum
43	2259	7	10.3	pg/mL	1	Serum
43	2260	7	7.88	pg/mL	1	Serum
43	2261	14	27.6	pg/mL	1	Serum
43	2262	14	187	pg/mL	1	Serum
43	2263	14	15.6	pg/mL	1	Serum
43	2264	14	34.9	pg/mL	1	Serum
43	2265	14	31.2	pg/mL	1	Serum
43	2266	14	14.2	pg/mL	1	Serum
43	2267	14	18.9	pg/mL	1	Serum
43	2268	14	25.2	pg/mL	1	Serum
43	2351	19	78.1	pg/mL	1	Serum
43	2352	19	30.8	pg/mL	1	Serum
43	2353	19	41.4	pg/mL	1	Serum
43	2354	19	28.7	pg/mL	1	Serum
43	2355	14	205	pg/mL	1	Serum
43	2356	19	89.2	pg/mL	1	Serum
43	2357	21	14.5	pg/mL	1	Serum
43	2358	19	221	pg/mL	1	Serum
43	2451	19	58.9	pg/mL	1	Serum
43	2452	14	21.9	pg/mL	1	Serum
43	2453	21	36.3	pg/mL	1	Serum
43	2454	14	56.6	pg/mL	1	Serum
43	2455	14	39.2	pg/mL	1	Serum
43	2456	14	20.8	pg/mL	1	Serum
43	2457	19	89.5	pg/mL	1	Serum
43	2458	14	33.3	pg/mL	1	Serum
33	2551	19	18.6	pg/mL	1	Serum
33	2552	19	47	pg/mL	1	Serum
33	2553	14	27.2	pg/mL	1	Serum
33	2554	19	33.4	pg/mL	1	Serum
33	2555	19	103	pg/mL	1	Serum
33	2556	21	43.4	pg/mL	1	Serum
33	2557	19	34.8	pg/mL	1	Serum
33	2558	21	6.91	pg/mL	1	Serum

Table 9: Sample Concentrations of IL-10 in Mouse Serum

Run ID	Subject	Day (Nominal)	Concentration (pg/mL)	Concentration Units	Dilution Factor	Biological Matrix
39	2051	3 Hr	7.37	pg/mL	1	Serum
39	2052	3 Hr	2.17	pg/mL	1	Serum
39	2053	3 Hr	2.61	pg/mL	1	Serum
39	2054	3 Hr	3.01	pg/mL	1	Serum
39	2055	3 Hr	3.78	pg/mL	1	Serum
39	2056	7	3.67	pg/mL	1	Serum
39	2057	7	3.89	pg/mL	1	Serum
39	2058	7	2.99	pg/mL	1	Serum
39	2059	7	4.72	pg/mL	1	Serum
39	2060	7	4.21	pg/mL	1	Serum
39	2061	14	5.46	pg/mL	1	Serum
39	2062	14	6.54	pg/mL	1	Serum
39	2063	14	7.39	pg/mL	1	Serum
39	2064	14	4.79	pg/mL	1	Serum
39	2065	14	6.29	pg/mL	1	Serum
39	2066	14	14.5	pg/mL	1	Serum
39	2067	14	3.34	pg/mL	1	Serum
39	2068	14	7.95	pg/mL	1	Serum
39	2151	3 Hr	4.31	pg/mL	1	Serum
39	2152	3 Hr	3.34	pg/mL	1	Serum
39	2153	3 Hr	3.45	pg/mL	1	Serum
39	2154	3 Hr	1.49	pg/mL	1	Serum
39	2155	3 Hr	1.99	pg/mL	1	Serum
39	2156	7	4.27	pg/mL	1	Serum
39	2157	7	3.57	pg/mL	1	Serum
39	2158	7	4.44	pg/mL	1	Serum
39	2159	7	4	pg/mL	1	Serum
39	2160	7	3.21	pg/mL	1	Serum
39	2161	14	7.59	pg/mL	1	Serum
39	2162	14	8.29	pg/mL	1	Serum
39	2163	14	159	pg/mL	1	Serum
39	2164	14	4.62	pg/mL	1	Serum
39	2165	14	8.26	pg/mL	1	Serum
39	2166	14	8.82	pg/mL	1	Serum
39	2167	14	9.16	pg/mL	1	Serum
44	2168	14	3.96	pg/mL	1	Serum
44	2251	3 Hr	3.04	pg/mL	1	Serum
44	2252	3 Hr	4.08	pg/mL	1	Serum
44	2253	3 Hr	2.29	pg/mL	1	Serum
44	2254	3 Hr	2.2	pg/mL	1	Serum

44	2255	3 Hr	2.05	pg/mL	1	Serum
44	2256	7	4.58	pg/mL	1	Serum

Table 9. continued

44	2257	7	35.7	pg/mL	1	Serum
44	2258	7	6.64	pg/mL	1	Serum
44	2259	7	3.44	pg/mL	1	Serum
44	2260	7	3.03	pg/mL	1	Serum
44	2261	14	12.1	pg/mL	1	Serum
44	2262	14	11.8	pg/mL	1	Serum
44	2263	14	4.74	pg/mL	1	Serum
44	2264	14	5.75	pg/mL	1	Serum
44	2265	14	4.55	pg/mL	1	Serum
44	2266	14	2.12	pg/mL	1	Serum
44	2267	14	4.71	pg/mL	1	Serum
44	2268	14	5.29	pg/mL	1	Serum
44	2351	19	16.9	pg/mL	1	Serum
44	2352	19	9.97	pg/mL	1	Serum
44	2353	19	6.54	pg/mL	1	Serum
44	2354	19	4.82	pg/mL	1	Serum
44	2355	14	15.3	pg/mL	1	Serum
44	2356	19	11.5	pg/mL	1	Serum
44	2357	21	2.29	pg/mL	1	Serum
44	2358	19	25.8	pg/mL	1	Serum
44	2451	19	37.6	pg/mL	1	Serum
44	2452	14	9.18	pg/mL	1	Serum
44	2453	21	6.19	pg/mL	1	Serum
44	2454	14	10.1	pg/mL	1	Serum
44	2455	14	11.9	pg/mL	1	Serum
44	2456	14	11.5	pg/mL	1	Serum
44	2457	19	14.7	pg/mL	1	Serum
44	2458	14	7.13	pg/mL	1	Serum
34	2551	19	12	pg/mL	1	Serum
34	2552	19	8.77	pg/mL	1	Serum
34	2553	14	5.85	pg/mL	1	Serum
34	2554	19	8.38	pg/mL	1	Serum
34	2555	19	14.2	pg/mL	1	Serum
34	2556	21	7.43	pg/mL	1	Serum
34	2557	19	11.2	pg/mL	1	Serum
34	2558	21	2.65	pg/mL	1	Serum

Table 10: Sample Concentrations of TNF-α in Mouse Serum

Run ID	Subject	Day (Nominal)	Concentration (pg/mL)	Concentration Units	Dilution Factor	Biological Matrix
40	2051	3 Hr	8.07	pg/mL	1	Serum
40	2052	3 Hr	4.04	pg/mL	1	Serum
40	2053	3 Hr	3.21	pg/mL	1	Serum
40	2054	3 Hr	5.72	pg/mL	1	Serum
40	2055	3 Hr	3.71	pg/mL	1	Serum
40	2056	7	6.34	pg/mL	1	Serum
40	2057	7	7.61	pg/mL	1	Serum
40	2058	7	4.16	pg/mL	1	Serum
40	2059	7	7.49	pg/mL	1	Serum
40	2060	7	9.22	pg/mL	1	Serum
40	2061	14	13.4	pg/mL	1	Serum
40	2062	14	10.3	pg/mL	1	Serum
40	2063	14	12.2	pg/mL	1	Serum
40	2064	14	18.3	pg/mL	1	Serum
40	2065	14	15.8	pg/mL	1	Serum
40	2066	14	29	pg/mL	1	Serum
40	2067	14	4.01	pg/mL	1	Serum
40	2068	14	22.3	pg/mL	1	Serum
40	2151	3 Hr	5.36	pg/mL	1	Serum
40	2152	3 Hr	3.67	pg/mL	1	Serum
40	2153	3 Hr	3.66	pg/mL	1	Serum
40	2154	3 Hr	2.36	pg/mL	1	Serum
40	2155	3 Hr	3.58	pg/mL	1	Serum
40	2156	7	8.99	pg/mL	1	Serum
40	2157	7	5.21	pg/mL	1	Serum
40	2158	7	7.72	pg/mL	1	Serum
40	2159	7	8.31	pg/mL	1	Serum
40	2160	7	5.97	pg/mL	1	Serum
40	2161	14	16.6	pg/mL	1	Serum
40	2162	14	19.6	pg/mL	1	Serum
40	2163	14	60	pg/mL	1	Serum
40	2164	14	13	pg/mL	1	Serum
40	2165	14	13	pg/mL	1	Serum
40	2166	14	24.8	pg/mL	1	Serum
40	2167	14	21	pg/mL	1	Serum
45	2168	14	16.1	pg/mL	1	Serum
45	2251	3 Hr	3.69	pg/mL	1	Serum
45	2252	3 Hr	9.65	pg/mL	1	Serum

45	2253	3 Hr	3.94	pg/mL	1	Serum
45	2254	3 Hr	3.41	pg/mL	1	Serum
45	2255	3 Hr	2.57	pg/mL	1	Serum
45	2256	7	9.93	pg/mL	1	Serum

Table 10. (continued)

45	2257	7	26.5	pg/mL	1	Serum
45	2258	7	10.4	pg/mL	1	Serum
45	2259	7	7.86	pg/mL	1	Serum
45	2260	7	4.06	pg/mL	1	Serum
45	2261	14	26.4	pg/mL	1	Serum
45	2262	14	40.4	pg/mL	1	Serum
45	2263	14	17.9	pg/mL	1	Serum
45	2264	14	18.7	pg/mL	1	Serum
45	2265	14	12.5	pg/mL	1	Serum
45	2266	14	9.24	pg/mL	1	Serum
45	2267	14	8.69	pg/mL	1	Serum
45	2268	14	15	pg/mL	1	Serum
45	2351	19	49.6	pg/mL	1	Serum
45	2352	19	38.9	pg/mL	1	Serum
45	2353	19	22.7	pg/mL	1	Serum
45	2354	19	19.9	pg/mL	1	Serum
45	2355	14	30.9	pg/mL	1	Serum
45	2356	19	21.1	pg/mL	1	Serum
45	2357	21	6.95	pg/mL	1	Serum
45	2358	19	58.8	pg/mL	1	Serum
45	2451	19	25.8	pg/mL	1	Serum
45	2452	14	23	pg/mL	1	Serum
45	2453	21	15.3	pg/mL	1	Serum
45	2454	14	31.3	pg/mL	1	Serum
45	2455	14	29.4	pg/mL	1	Serum
45	2456	14	30.3	pg/mL	1	Serum
45	2457	19	43.2	pg/mL	1	Serum
45	2458	14	16.9	pg/mL	1	Serum
35	2551	19	21.4	pg/mL	1	Serum
35	2552	19	21.7	pg/mL	1	Serum
35	2553	14	17.8	pg/mL	1	Serum
35	2554	19	15.5	pg/mL	1	Serum
35	2555	19	24.3	pg/mL	1	Serum
35	2556	21	19.8	pg/mL	1	Serum
35	2557	19	24.1	pg/mL	1	Serum
35	2558	21	3.93	pg/mL	1	Serum
